# Simulating EGFR-ERK Signaling Control by Scaffold Proteins KSR and MP1 Reveals Differential Ligand-Sensitivity Co-Regulated by Cbl-CIN85 and Endophilin

**DOI:** 10.1371/journal.pone.0022933

**Published:** 2011-08-01

**Authors:** Lu Huang, Catherine Qiurong Pan, Baowen Li, Lisa Tucker-Kellogg, Bruce Tidor, Yuzong Chen, Boon Chuan Low

**Affiliations:** 1 Computational Systems Biology, Singapore-MIT Alliance, National University of Singapore, Singapore, Singapore; 2 Department of Pharmacy, National University of Singapore, Singapore, Singapore; 3 Department of Biological Engineering, Department of Electrical Engineering & Computer Science, Computer Science and Artificial Intelligence Laboratory, Massachusetts Institute of Technology, Cambridge, Massachusetts, United States of America; 4 Singapore-MIT Alliance for Research and Technology, Singapore, Singapore; 5 Department of Biological Sciences, National University of Singapore, Singapore, Singapore; 6 Department of Physics, National University of Singapore, Singapore, Singapore; 7 Mechanobiology Institute, National University of Singapore, Singapore, Singapore; Ohio State University, United States of America

## Abstract

ERK activation is enhanced by the scaffolding proteins KSR and MP1, localized near the cell membrane and late endosomes respectively, but little is known about their dynamic interplay. We develop here a mathematical model with ordinary differential equations to describe the dynamic activation of EGFR-ERK signaling under a conventional pathway without scaffolds, a KSR-scaffolded pathway, and an MP1-scaffolded pathway, and their impacts were examined under the influence of the endosomal regulators, Cbl-CIN85 and Endophilin A1. This new integrated model, validated against experimental results and computational constraints, shows that changes of ERK activation and EGFR endocytosis in response to EGF concentrations (i.e ligand sensitivity) depend on these scaffold proteins and regulators. The KSR-scaffolded and the conventional pathways act synergistically and are sensitive to EGF stimulation. When the KSR level is high, the sensitivity of ERK activation from this combined pathway remains low when Cbl-CIN85 level is low. But, such sensitivity can be increased with increasing levels of Endophilin if Cbl-CIN85 level becomes high. However, reduced KSR levels already present high sensitivity independent of Endophilin levels. In contrast, ERK activation by MP1 is additive to that of KSR but it shows little ligand-sensitivity under high levels of EGF. This can be partly reversed by increasing level of Endophilin while keeping Cbl-CIN85 level low. Further analyses showed that high levels of KSR affect ligand-sensitivity of EGFR endocytosis whereas MP1 ensures the robustness of endosomal ERK activation. These simulations constitute a multi-dimensional exploration of how EGF-dependent EGFR endocytosis and ERK activation are dynamically affected by scaffolds KSR and MP1, co-regulated by Cbl-CIN85 and Endophilin A1. Together, these results provide a detailed and quantitative demonstration of how regulators and scaffolds can collaborate to fine-tune the ligand-dependent sensitivity of EGFR endocytosis and ERK activation which could underlie differences during normal physiology, disease states and drug responses.

## Introduction

The duration, magnitude and sub-cellular compartmentalization of ERK activation elicits different cellular outcomes leading to functional activation, proliferation, differentiation, migration, or survival [1], [2]. For instance, in PC12 cells, sustained ERK activation causes differentiation [3], [4], strong ERK activation leads to differentiation in normal cells and survival in carcinoma cells, whereas weak ERK activation results in proliferation in normal cells and apoptosis in carcinoma cells [5]. These outcomes are collectively regulated by a number of regulators under different physiological conditions [1], [5] and disease states, such as tumorigenesis [6], cardiovascular disease [7], [8], and urinary bladder dysfunction [9].

One important class of ERK regulators are scaffold proteins that compartmentalize and spatio-temporally control ERK signaling to regulate signaling strength and duration, confer signaling specificity, diversify signaling kinetics, and prevent signaling activation by irrelevant stimuli [10], [11], [12]. Scaffold proteins perform these tasks by assembling signaling components, localizing signaling molecules, coordinating positive and negative feedback, and insulating activated signaling molecules from inactivation. Two scaffold proteins, Kinase Suppressor of Ras (KSR1) and MEK Partner 1 (MP1), are involved in the regulation of EGF-induced ERK signaling in PC12 [13], [14] and other cells [10], [11]. KSR is a multi-domain protein that binds Raf-1, MEK, ERK, and several other proteins. In resting cells, it is sequestered in the cytosol by 14-3-3 proteins. In response to EGF stimulation, KSR is released from 14-3-3 and recruited to the plasma membrane to scaffold Raf-1, MEK1/2 and ERK1/2 and to subsequently facilitate Ras activation of the Raf-MEK-ERK module [10], [11]. On the other hand, MP1 is a widely expressed small scaffold protein that is recruited to late endosomes by the adaptor protein p14, where it promotes the assembly and interaction of MEK1 and ERK1. Upon stimulation by the internalized activated cell surface receptors that are trafficked to the late endosomes [14], MP1 facilitates Ras activation of the MEK1-ERK1 module there [10], [11], [14], [15].

Some important aspects and functional implications of the collective actions of these two scaffold proteins on ERK signaling have been studied. It has been suggested that sustained ERK activation may require coordinated control by KSR and the MP1-p14 complex to facilitate continued signaling from the plasma membrane to late endosomes [16], with KSR supporting the proliferative and transforming functions of ERK signaling and MP1 converting low MEK activity into sustained ERK activation [10], [17]. Overexpression of both MP1 and KSR can lead to different responses, depending on the relative stoichiometry of the individual components [18]. For instance, overexpression of B-KSR in PC12 cells, a neuronal-specific isoform of KSR, switches EGF signaling from a brief proliferative signal to a sustained differentiation signal [13]. Because endosomes are immediately derived from the plasma membrane compartment, MP1-mediated signaling serves as an extension of KSR-mediated signaling at the cell membrane and maintains signaling at an adequate strength and duration, and in some cases with qualitatively different signaling kinetics, upon the removal of the activated receptors from the cell membrane [11]. Furthermore, it enables the regulation of endosomal traffic and cellular proliferation during tissue homeostasis [15].

KSR appears to play important roles in the regulation of adipogenesis [19], neuronal differentiation and functioning [13], Ras-mediated cancer formation, susceptibility toward rheumatoid arthritis [20], and cellular sensitivity to anticancer agents [21]. KSR also regulates the response of intestinal epithelial cells during inflammation and inflammatory bowel disease via activation of cell survival pathways [22], [23]. In comparison, the MP1-p14 complex is required in prostate cancer cell migration [24] via PAK1-dependent ERK activation during adhesion and cell spreading [25], [26].

Despite such functional significance, the detailed dynamics and functional consequences of the coordinated actions of KSR and MP1 remain to be fully elucidated [15]. Quantitative study of the effects by these scaffold proteins and other regulators on ERK signaling is useful for facilitating more comprehensive study and understanding whether they exert their roles in isolation or in concert during cell signaling and dynamics, disease manifestation and drug responses. To this end, a mathematical model of the MAPK cascade with a generic scaffold protein was developed and has shown its capability in a quantitative analysis of the effects of scaffold–kinase complexes in regulating the specificity, efficiency, and amplitude of MAPK signal propagation (e.g., the levels of biphasic MAPK activation and the threshold of altered MAPK activation) [27], [28]. This model can be combined with established mathematical models of the EGFR-ERK pathway [29], [30], [31], [32], [33], [34] and those coupled with such regulators as sprouty [32], Rap1 [33], and MEKK1 [35] to further examine the collective effects of these scaffold proteins and other regulators on ERK signaling.

In normal and disease conditions, the circulating level of epidermal growth factor (EGF) is mostly not constant, either caused by fluctuation of growth factors or receptor binding. This concentration fluctuation might be essential for cell development and tissue repair [36], since a number of previous studies have reported that the production and secretion variation of growth factors such as EGF during different developmental stages might exert a profound effect on tissue cell growth and differentiation [37], [38], [39]. During tumor cell invasion, elevated expression of EGF has been found to diffuse and generate a gradient of EGF receptor activation in adjacent cells, leading to an increase in tumor cell motility and invasiveness, thereby enhancing cancer cell metastasis [40], [41].

EGFR is activated through binding of EGF, followed by internalization into early endosomes from where it is either recycled to the plasma membrane or sorted to late endosomes for degradation [42], [43], [44]. Although endocytosis has traditionally been viewed simply as diminishing EGFR signaling [45], evidence of increased tyrosine phosphorylation of endosomal EGFRs and their association of Shc and Grb2 indicates that endocytosis can temporally and spatially regulate the signaling cascades [46], [47], [48], [49]. An important step in initiating EGFR internalization is the binding of adaptor protein CIN85 to the ubiquitin ligase c-Cbl complex, which subsequently recruits activated EGFR on the plasma membrane [50], [51]. The complex then associates with other proteins such as Endophilin A1, dynamin-2, synaptojanin and amphiphysin to drive clathrin assembly and EGFR endocytosis [52]. However, in PC12 cells, active RhoA effector ROCK phosphorylates Endophilin A1 and inhibits the recruitment of Endophilin A1 to the EGFR–c-Cbl–CIN85 complex, thereby reducing the level of EGFR endocytosis [53]. These results raise the possibility that both Cbl and Endophilin A1 could play an important role in coordinating Ras/ERK signaling. However, whether their effects are linked to scaffold functions of KSR or/and MP1 remains unknown.

Based on our previous mathematical model of the EGFR-ERK pathway [35], [54] and that of the MAPK cascade with generic scaffold proteins [27], [28], we here report a mathematical model of the EGFR-ERK pathway in PC12 cells that includes the two scaffold proteins KSR and MP1. Using this model, we examine, for the first time, how the scaffolds could modulate the robustness and sensitivity of Ras/ERK under the influence of varying extracellular EGF concentrations and two intracellular regulators downstream of EGFR, the Cbl-CIN85 and Endophilin A1. In this integrated pathway model, ordinary differential equations were used to represent the time-dependent dynamic behavior of the concentration of proteins and other molecules and the kinetics of their interactions in the pathway. Our simulation model was validated by measuring the agreement with a number of experimental findings and previous simulation results for the effects of various perturbations (EGF, PP2A, MKP3, KSR, MP1, and p14) on ERK activities. Simulating the collective effect of KSR and MP1 on ERK activation revealed that KSR acts synergistically with the conventional EGFR-Ras-Raf-MEK-ERK module to elicit acute ERK activation which is also sensitive to changes in the EGF concentrations. In contrast, MP1 appears to act in parallel (additively but not synergistically) with KSR for the chronic ERK activation which is not responsive to changes in the EGF concentrations unless by increasing level of Endophilin A1 while keeping the level of Cbl-CIN85 low. By comparing the sensitivity of endocytosed EGFR and the sensitivity of activated endosomal ERK, our simulations further reveal that KSR and MP1 exert differential impacts on these two responses. Changes to the ERK sensitivity appear to be more gradual and “analog-like” than those for the endocytosed EGFR (more “digital-like) if KSR was present in optimally high levels. Furthermore, MP1 appears to maintain more robust endosomal ERK activation than for the endocytosed EGFR.

Therefore, the apparent difference in their ligand-sensitivity could be influenced not just by the scaffolds alone but most likely via their relative concentrations and interplay with other immediate regulators such as the Cbl-CIN85 and Endophilin A1. All these results point to the importance of understanding the functional interplay between compartment-specific scaffolds and other immediate regulators in ensuring ligand-sensitivity of Ras/ERK signaling. Such responses could underlie the differences during normal physiological and pathophysiological conditions as well as during drug treatments.

## Results and Discussion

### Constructing a new mathematical model of EGFR-ERK signaling with key scaffolds and regulators

**Figure 1:**
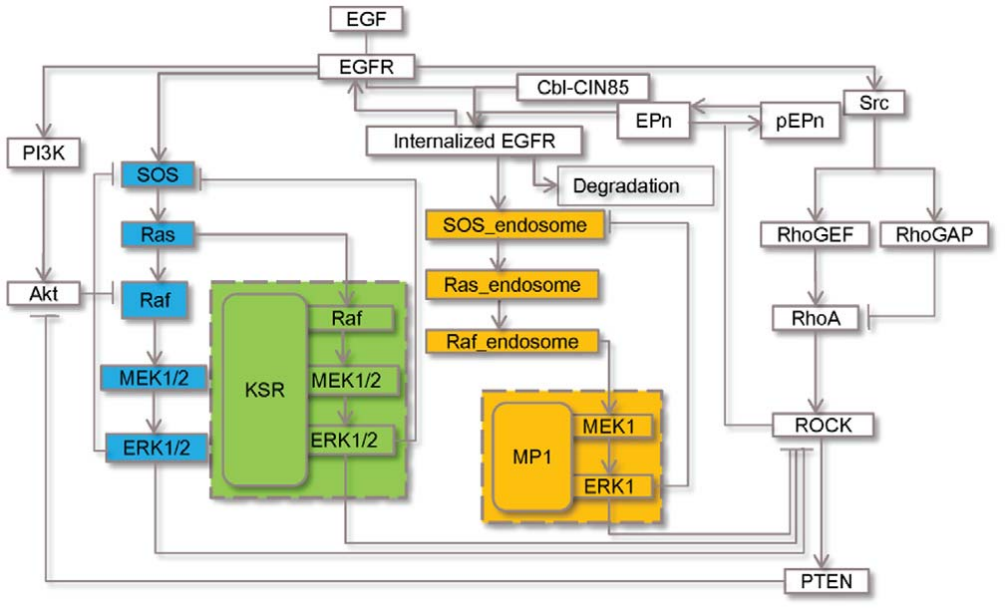
A model for Ras/ERK signaling pathway regulated by scaffolds and modulators. More detailed biochemical model of scaffolding actions of KSR and MP1 in the dashed rectangular boxes are shown in Figures 2 and 3, respectively. Molecules highlighted in *blue*, *green* and *orange boxes* represent the separate modules of Ras/ERK signaling operating from the conventional mode (no-scaffolds; membrane), KSR-supported mode (membrane) and MP1-supported mode (late endosome), respectively.

**Figure 2:**
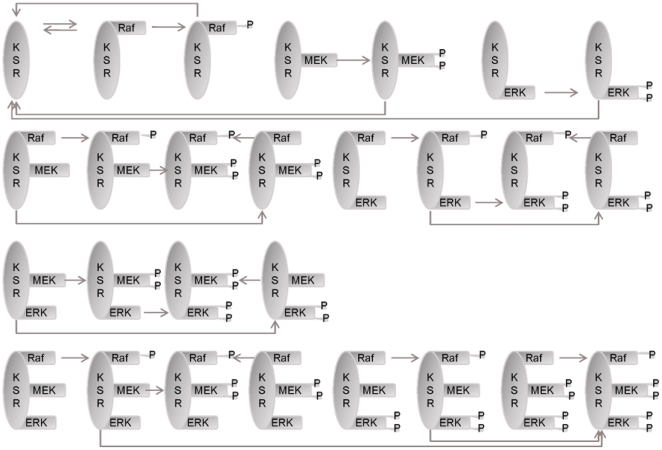
A detailed biochemical model of scaffolding action of KSR. Please refer to “Methods” for the considerations and assumptions used and Supplementary Table S1 for detailed descriptions of the kinetics parameters. “P” denotes protein phosphorylation.

To examine the impacts by the two compartment-specific scaffolds KSR and MP1 on Ras/ERK activity under the influence of Cbl and Endophilin A1 and varying EGF concentrations, we have constructed a new mathematical model by integrating these molecular species as depicted in **Figure 1**. It takes into consideration the biochemical model of scaffolding actions by KSR and MP1 (**Figures 2** **and** **3**, respectively) which are based on previous models of the MAPK cascade with generic scaffold proteins [27], [28]. Detailed molecular interactions and the corresponding kinetic data were obtained from the published simulation models and further literature, summarized in **Supplementary Table S1**. Toward validating the model, we examined whether the results are consistent with experimental observations. The results in **Supplementary Figures S1 and S2** show that at 100 ng/ml EGF, the simulated ERK activation peaks at ∼5 minutes and decays within 50 minutes. This is consistent with the observation that treatment of 100 ng/ml EGF in PC12 cells transiently activates ERK, which peaks within 5 minutes and thereafter it decays within 30–60 minutes [33], [55]. Upon EGF stimulation, SOS is recruited to the plasma membrane where it activates Ras, switching inactive GDP-bound Ras into active GTP-bound form, and recruits the Raf kinase to the plasma membrane, initiating the signaling cascades. Similarly, our simulation shows that the amount of active RasGTP peaks at ∼2.5 minutes and quickly it decays within 20 minutes, consistent with the observation that active RasGTP levels in EGF-treated PC12 cells increase dramatically within 5 minutes and decay steeply within 10 minutes [33].

**Figure 3:**
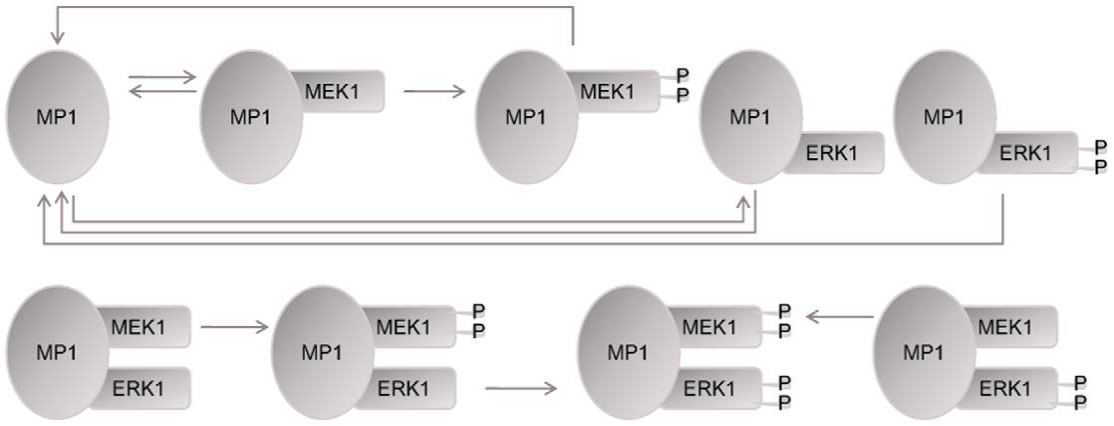
A detailed biochemical model of scaffolding action of MP1. Please refer to “Methods” for the considerations and assumptions used and Supplementary Table S1 for detailed descriptions of their kinetics parameters. “P” denotes protein phosphorylation.

### Simulation models on KSR and MP1/p14-mediated pathways

**Figure 4:**
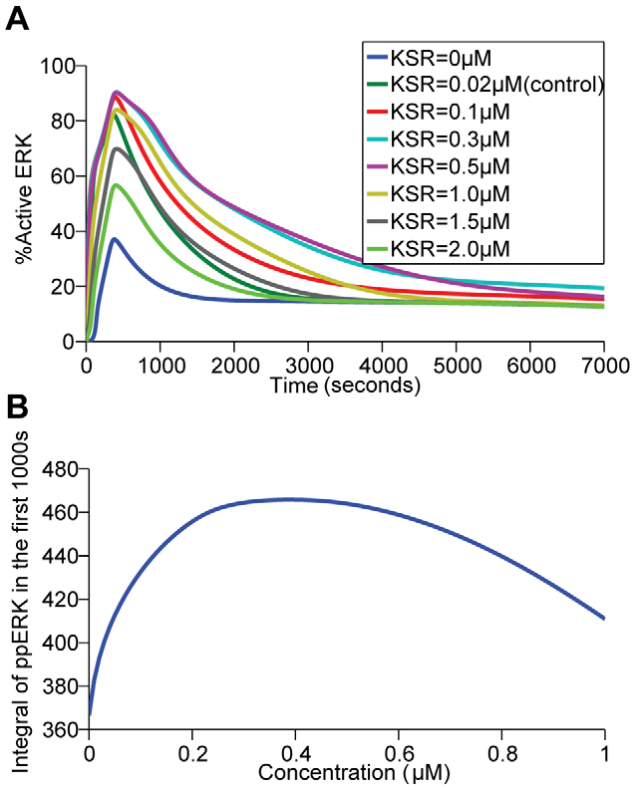
The biphasic effect of KSR on ERK activation. (A) Percentage of active ERK was plotted over the period indicated by varying the concentrations of KSR from 0 to 2 µM. (B) Existence of an optimal scaffold concentration of KSR (0.3–0.5 µM) by plotting the time integral of ppERK/ERK in the first 1000 seconds and KSR's initial concentrations.

Recent studies have identified the double effects of MAPK scaffold proteins KSR and MP1 on ERK activation, with the hallmark of either promoting or inhibiting the signals depending on their local concentrations. The promoting effect is due to the ability of the scaffolds to recruit the proteins to a limited number of locations which each has high concentration of partner proteins. However, an excessive number of locations where each has low concentration would sequester the individual protein partners from reaching each other [56], [57], [58]. The kinetics of KSR-mediated signaling was validated by evaluating the effect of altered KSR concentration on ERK activation. When KSR1 was experimentally re-introduced into KSR1 ^−/−^ mouse embryonic fibroblasts, it demonstrates a biphasic effect on ERK signaling, such that signaling is increased in a concentration-dependent manner when KSR concentration is increased up to 14 fold of wild-type levels. However, further increase of KSR concentration leads to decreased ERK signaling [59]. Such distinct biphasic effect of KSR is demonstrated in our simulation results (**Figure 4A and 4B**) such that at low concentrations, KSR has a positive effect on ERK activation; while at high concentrations, it negatively regulates ERK activation.

**Figure 5:**
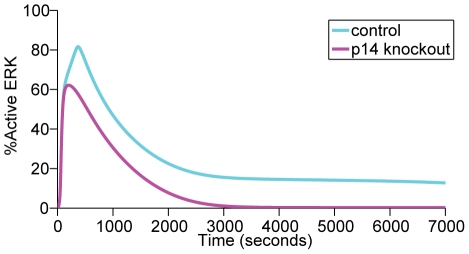
Profile of ERK activation before and after p14 knockout. The amount of active ERK produced in the later phase (>600 seconds) is reduced.

Similarly, the kinetics of MP1-mediated signaling was also validated by evaluating the effect of altered concentrations of MP1 adaptor protein p14 on ERK activation. As shown in **Figure 5**, p14 “knockout” only affects the later phase of activated ERK dynamics, resulting in a decrease in the duration of ERK activation. This result is consistent with the observation that the MP1-p14 complex is not required for initial signaling near the plasma membrane but is necessary for the activation at endosomes 10–30 min following EGF treatment in Hela cells [60]. This illustrates the intricate mechanism that exists in a given cell allowing the MAPK pathway to be activated with different kinetics through localized scaffold proteins, an essential feature of compartmentalized signaling.

**Figure 6:**
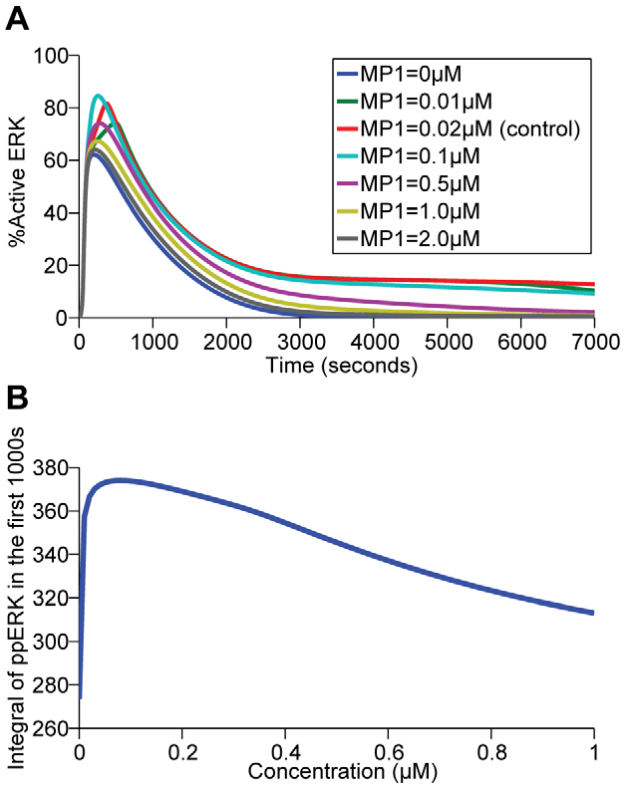
The biphasic effect of MP1 on ERK activation. (A) Percentage of active ERK was plotted over the period indicated by varying the concentrations of MP1 from 0 to 2 µM. (B) Existence of an optimal scaffold concentration of MP1 (0.1 µM) by plotting the time integral of ppERK/ERK in the first 1000 seconds and MP1's initial concentrations.

Moreover, the scaffold-specific biphasic property of MP1 can be observed through the simulated plot here, as consistent with a reported result that higher level of MP1 will lead to inhibition of signaling (**Figure 6**) [61]. The findings that the expression level of scaffold protein in wildtype cells is sub-optimal for signaling, may provide regulatory flexibility as tuning scaffold protein expression up or down directly modulates the downstream phenotypic response.

To further validate how the current model operates in the presence of other signaling nodes, we evaluated the significance of phosphatases PP2A and MKP3 on ERK activation (**Supplementary Figures S3 and S4**). As a result, variation of PP2A at low concentrations from 0.005 to 0.01 µM showed little effect on the maximal ERK activation but they reduced the rate of its decay. Similarly, at lower levels, variation of MKP3 levels from 0.0005 to 0.001 µM had little effect on the maximal ERK activation but they also reduced the rate of its decay [62]. In contrast, at higher levels of PP2A and MKP3, both the maximal amount and duration of ERK activation had decreased. Taken together, our newly refined model recapitulates core signaling dynamics observed in the presence of KSR and MP1 and is ready to interrogate how they would function independently or collectively in various EGF regimes as further detailed below.

### Collective effects of KSR and MP1 on ERK activation

**Figure 7:**
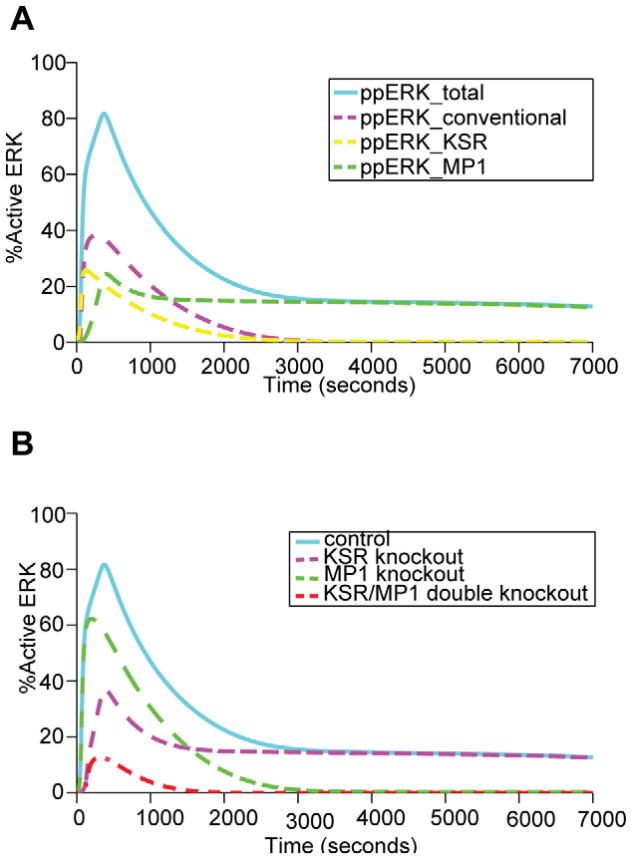
The collective effect of scaffold proteins KSR and MP1 on ERK activation. (A) Overall signaling and contribution from individual modules. (B) Signaling profile under knockout conditions.

Next, the effect of KSR and MP1 on ERK activation was simulated under the condition without these scaffolds (conventional) or with their presence, either separately or together. The results show that both KSR and MP1 increased the level of acute ERK activation within 2000 s, and with only the MP1 contribution, the signal was maintained for more than 2 hours (**Figure 7A**). This simulation result is consistent with the experimental indication that sustained ERK activation may arise from collective action of KSR and MP1 [16]. The slight difference between MP1-mediated ERK activation profile (**Figure 7A**) and KSR-knockout ERK activation profile (**Figure 7B**) is mainly due to the contribution from the conventional (un-scaffolded) pathway for producing the remaining active ERK.

However, under MP1-knockout condition, there is a moderate reduction in the level of ERK activation at short times of up to 2000 s, but it completely eliminated the sustained activation of ERK at times beyond 3000 s. In strong contrast, KSR-knockout significantly reduced the level of ERK activation at short times of up to 2000 s, but ERK activation was not reduced at times beyond 3000 s (**Figure 7B**). This is also consistent with the experimental indication that KSR supports the proliferative and transforming functions of ERK, and MP1 converts low MEK activity into sustained ERK activation [10], [17]. While the results strongly indicate that both scaffolds contribute to the majority, if not all of ERK activation in the current model, it remains unclear whether they exert their effects in parallel (additively) or in synergism or whether both pathways are subjected to fluctuations of EGF concentrations. To examine this, we went on to simulate ERK dynamics by KSR and/or MP1, separately or together when subjected to varying concentrations of EGF as described below.

Quantitatively, our simulations suggested that MP1 knockout reduces the peak amplitude of ERK activation by 25%, which is consistent with the observed 30% reduction of ERK activation by the loss of function of p18 that excludes the p14-MP1 complex from late endosomes [14]. Our simulations also predicted that KSR knockout would reduce the peak amplitude of ERK activation by 50%, which is consistent with the observation that ERK activation in response to multiple stimuli was attenuated but not abolished in the KSR^−/−^ mouse embryo fibroblasts [17]. As strong and transient ERK activation is required for the proliferation of PC12 cells [5], the reduced peak/amplitude in the KSR knockout is expected to significantly limit the proliferation processes. This is consistent with the experimental finding that loss of KSR1 expression attenuated ERK signaling and abolished the capability of oncogenic Ras to induce skin cancer in KSR^−/−^ mice [63]. Moreover, our simulation suggested that double knockout of KSR and MP1 significantly reduced the strength and duration of ERK activation with the peak being reduced by 8-fold.

### Synergistic ERK activation by the conventional module and KSR-mediated module

**Figure 8:**
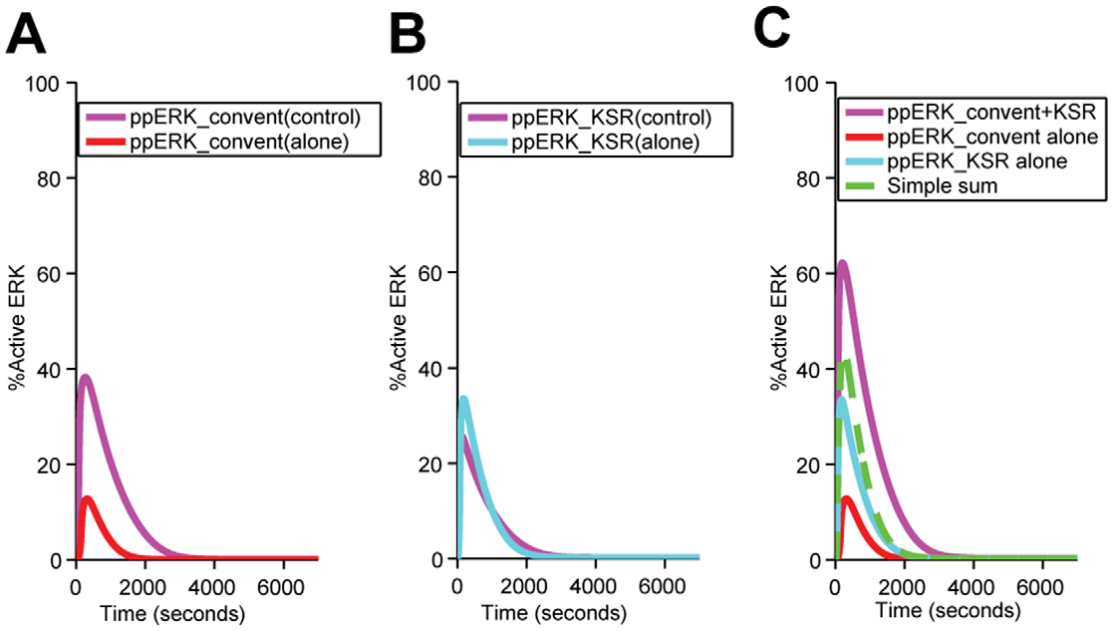
The synergistic effect of the two membrane associated signaling components, the conventional EGFR–Ras–Raf–MEK–ERK signaling module (Convent) and the KSR-mediated module (KSR) on ERK activation.

Experimental [64], [65] and computational [28] studies have shown that, due to its scaffolding activities, KSR enhances the efficiency of ERK activation without altering the fundamental system outputs, i.e. the incoming signals are amplified or attenuated in different biological contexts and at different KSR concentrations. Underlying this fundamental consistency is a complex interplay between conventional pathway and pathways mediated by scaffolds. Based on models of the MAPK cascade with generic scaffold proteins [27], [28], shown in **Figure 2** **and** **3**, KSR at cell membranes releases activated signaling molecules and competes with the conventional unscaffolded pathway for inactive signaling molecules. The former action enhances and the latter action reduces the capability of the conventional pathway for ERK activation. If the former action outweighs the latter, then KSR is expected to enhance ERK activation not only by its own signaling but also by synergistically increasing the signaling of the conventional unscaffolded pathway. The contribution of the conventional pathway with and without KSR (**Figures 8A**) and the KSR-mediated pathway with and without the conventional route of ERK activation (**Figure 8B**) were compared. The results show that the level of ERK activation arising from signaling via the conventional pathway in the presence of KSR is significantly increased with respect to that without KSR (**Figure 8A**) whereas the level of ERK activation arising from signaling via the KSR-mediated pathway in the presence of the conventional one is slightly decreased when compared to that without the conventional pathway (**Figure 8B**). Consistently, **Figure 8C** shows the synergistic effect of the conventional and KSR-mediated pathways on ERK activation. Therefore, our simulation study suggested that the signal-enhancing action of KSR on the conventional pathway significantly outweighs its signal-reducing action on the conventional module, leading to a significantly stronger combined signaling from the two membrane modules than the simple sum of each individual component. This synergistic effect may enable sizable ERK activation at moderate or suboptimal (which is below the concentration for the maximum total amount of active ERK produced in the first 1000 seconds across 0–1.0 µM scaffold concentration, **Figure 4B** **and** **6B**) levels of KSR in many cells [21].

### Distinct signaling dynamics of the membrane and late endosome components in response to varying EGF levels

**Figure 9:**
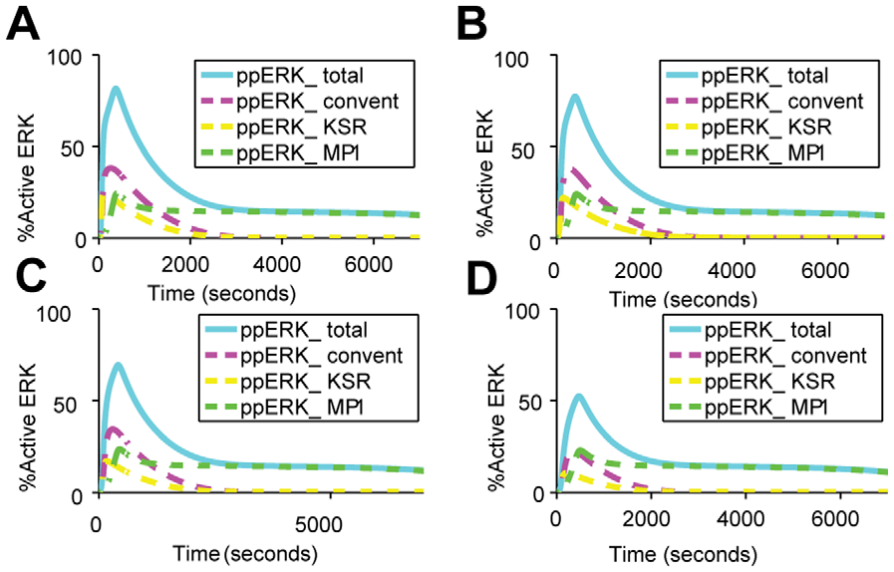
ERK activation regulated by membrane- and endosome-based modules. The contribution of the two membrane and one endosome components, the conventional EGFR–Ras–Raf–MEK–ERK signaling module (Convent), the KSR-mediated module (KSR), and the MP1 module (MP1), on ERK activation at various EGF concentrations: (A) 100 ng/ml, (B) 60 ng/ml, (C) 40 ng/ml, (D) 25 ng/ml.

Since under various physiological conditions, concentrations of growth factors are more likely to change and present in a gradient instead of being constant, we set out to examine whether there exists any significant perturbations in the signaling dynamics of the membrane and late endosomal components in response to varying EGF levels. **Figure 9** shows the contribution of the membrane (conventional and KSR-mediated pathways) and late endosome (MP1-mediated pathway) components, on ERK activation at EGF concentrations ranging from 25 ng/ml to 100 ng/ml. Interestingly, the signaling via the late endosomal component is insensitive to the variation of EGF concentrations under this condition. In contrast, signaling through the two membrane components is substantially altered by varying EGF doses, specifically when EGF is reduced from 40 ng/ml to 25 ng/ml. **Supplementary Figure S5** further shows the relative sensitivity of these two sub-pathways at high EGF dose – the sensitivity of membrane subpathway (conventional and KSR-mediated one) is almost 4 times the sensitivity of endosomal subpathway. These results are consistent with the prediction using principle component analysis that receptor internalization and endosomal signaling are important features regulating signal output at lower EGF doses [66].

### The compartment-specific sensitivity toward EGF variations is co-regulated by endocytosis proteins and scaffold proteins

**Figure 10:**
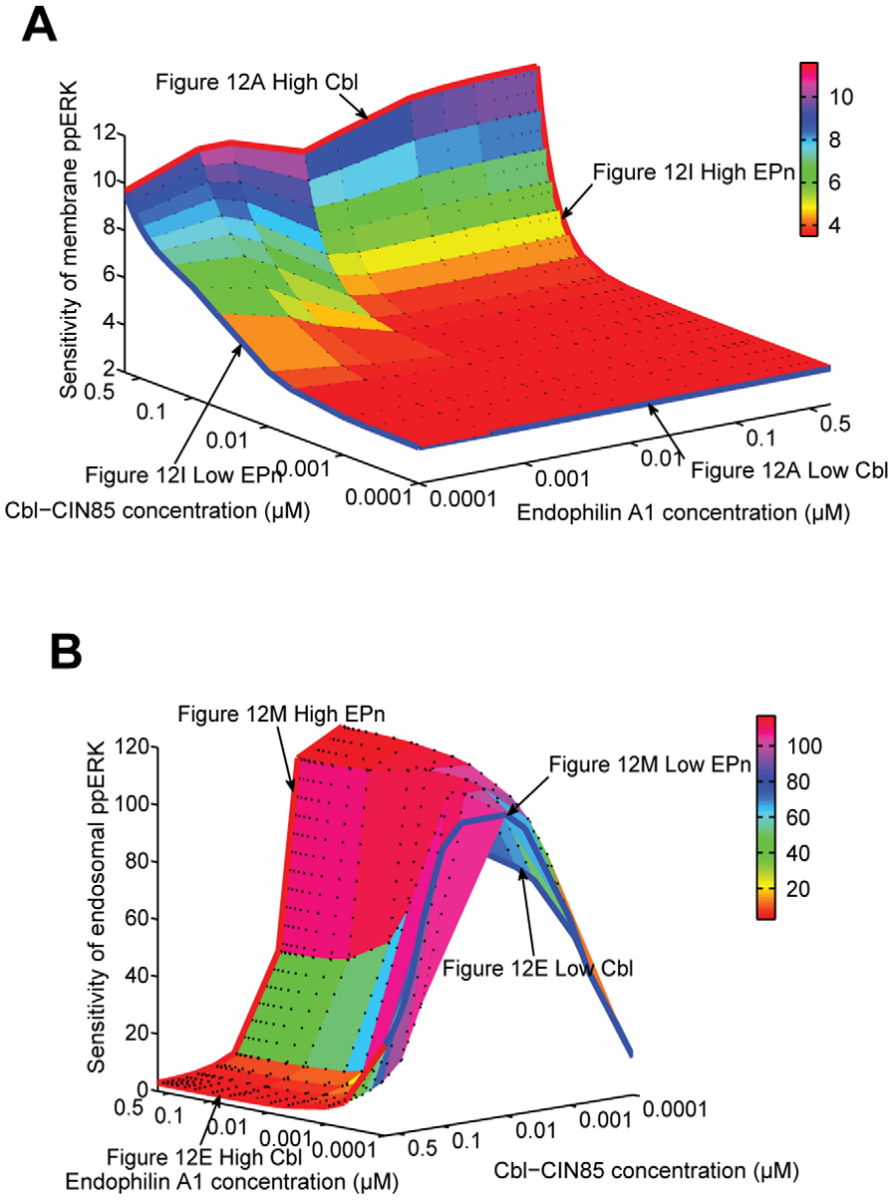
Differential sensitivity of ppERK from (A) membrane and (B) endosomal subpathways for EGF under various Cbl-CIN85 and Endophilin A1 concentrations when both scaffold proteins KSR and MP1 are expressed at sub-optimal “low” levels. For clarity, the sensitivity levels are denoted by colors in the scale bars. Arrows indicate response curves under the conditions specified and detailed in Figure 12, Panels A, E, I, M.

**Figure 11:**
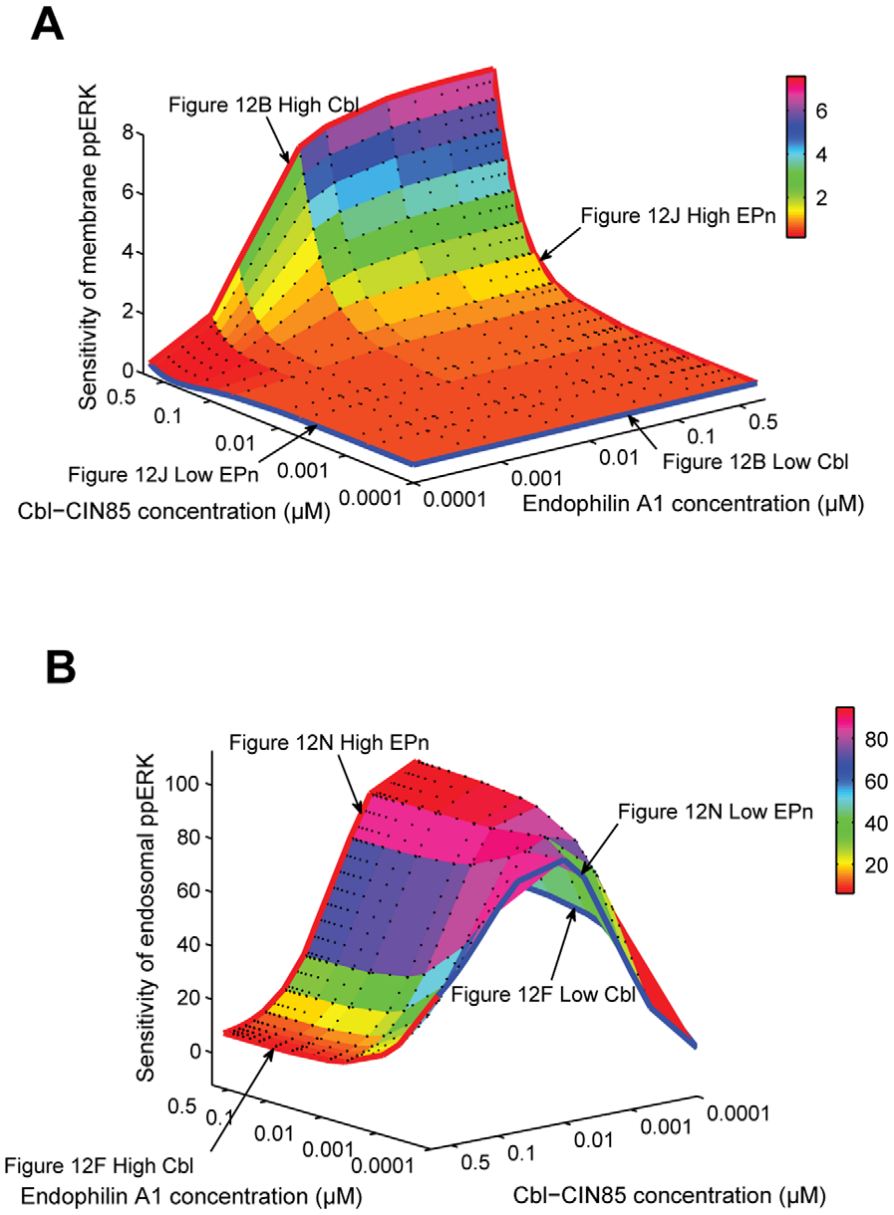
Differential sensitivity of ppERK from (A) membrane and (B) endosomal subpathways for EGF under various Cbl-CIN85 and Endophilin A1 concentrations when scaffold proteins KSR and MP1 are highly expressed – optimal for signaling. For clarity, the sensitivity levels are denoted by colors in the scale bars. Arrows indicate response curves under the conditions specified and detailed in Figure 12, Panels B, F, J, N.

**Figure 12:**
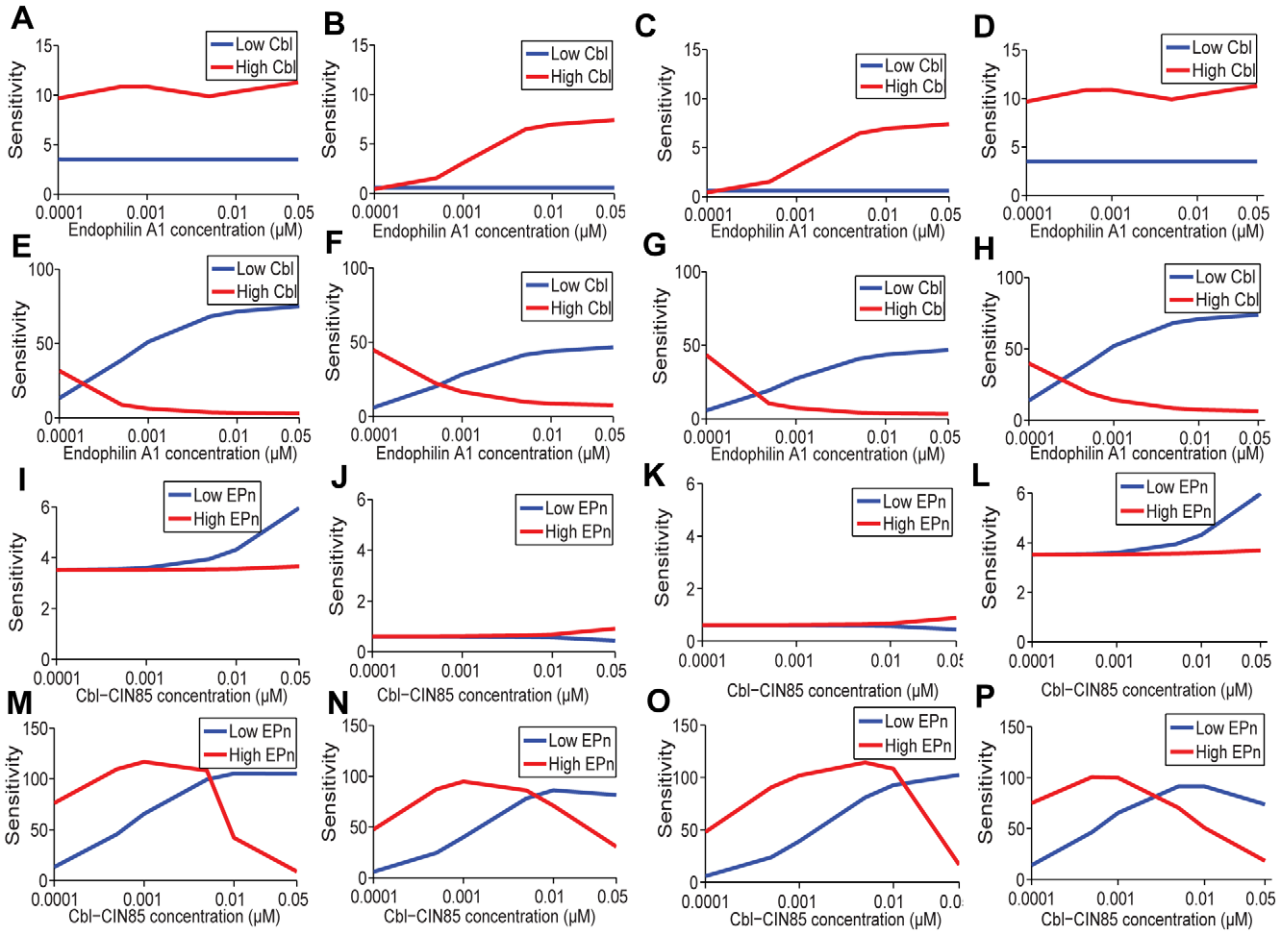
Detailed analysis on the sensitivities of activated ERK mediated by scaffold proteins KSR and MP1 and modulators Cbl-CIN85 and Endophilin A1 under various situations. Sensitivities of first and third panel (A, B, C, D, I, J, K, L) KSR-mediated and conventional; second and fourth panel (E, F, G, H, M, N, O, P) MP1-mediated subpathways toward EGF variation regulated by (A - H) Endophilin A1 concentration variation when Cbl-CIN85 is low (0.0001 µM) or high (0.8 µM); (I - P) Cbl-CIN85 concentration variation when Endophilin A1 is low or high at four conditions: (A, E, I, M) when both scaffolds are present in suboptimal “low” levels [KSR  = 0.02 µM, MP1  = 0.02 µM]; (B, F, J, N) when both scaffolds are present in optimally “high” concentrations as determined earlier [KSR  = 0.3 µM, MP1  = 0.3 µM]; (C, G, K, O) when KSR is present at “high” level [0.3 µM] and MP1 at “low” level [0.02 µM]; (D, H, L, P) when MP1 is present at “high” level [0.3 µM] and KSR is at “low” level [0.02 µM].

Through ligand-induced receptor activation, any changes in the EGF concentrations could lead to altered levels of activated EGFR on the cell surface, thus affecting its downstream signaling via both the membrane components (the conventional and KSR-mediated pathway) and the late endosomal component (MP1-mediated pathway). We therefore hypothesize that the apparent difference in their ligand-sensitivity could be influenced not just by the scaffolds alone but most likely via their relative concentrations and interplay with other immediate regulators such as the Cbl-CIN85 and Endophilin A1. To this end, we conducted further simulations by varying concentrations of Cbl-CIN85 and Endophilin A1 and tested their impacts on the ligand sensitivity mediated by the membrane (conventional plus KSR; or the endosomal module (MP1) under the following 4 conditions: (1) when both scaffolds are present in suboptimal “low” levels [KSR  = 0.02 µM, MP1  = 0.02 µM] (**Figures 10**), (2) when both scaffolds are present in optimally “high” concentrations as determined earlier [KSR  = 0.3 µM, MP1  = 0.3 µM] (**Figures 11**), (3) when KSR is present at “high” level [0.3 µM] and MP1 at “low” level [0.02 µM] (**Figure 12**), and (4) when MP1 is present at “high” level [0.3 µM] and KSR is at “low” level [0.02 µM] (**Figure 12**). Results of sensitivity analyses in **Supplementary Figure S5** show that the MP1-scaffolded module bears little sensitivity in response to varying EGF doses under condition (1) when both scaffolds are present in suboptimal “low” levels [KSR  = 0.02 µM, MP1  = 0.02 µM].

The KSR-scaffolded pathway and the conventional pathway are sensitive to EGF stimulation and their combined effects on ERK activation are synergistic. When the KSR level is high, the sensitivity of this combined pathway remains low in the presence of low concentration of Cbl-CIN85 while such sensitivity can be increased with increasing levels of Endophilin A1 if the amount of Cbl-CIN85 becomes high (**Figure 12** **, Panels B**, **C**). However, reduced KSR level already presents high sensitivity that is independent of the levels of Endophilin A1 (**Figure 12**, **Panels A**, **D**). In contrast, the ERK activation by MP1-scaffolded pathway is additive to that of KSR but it shows little ligand-sensitivity under high levels of EGF stimulation. Such inert sensitivity can, however, be reversed in part by increasing level of Endophilin A1 while keeping the level of Cbl-CIN85 low (**Figure 12**, **Panels E**, **F**, **G**, **H**) or by increasing level of Cbl-CIN85 while keeping the level of Endophilin A1 low (**Figure 12**, **Panels M**, **N**, **O**, **P**). Thus, this current study extends the observations of others [31], thereby suggesting that the process of endocytosis plays a prominent role in regulating signal output sensitivity in response to different EGF dosages.

**Figure 13:**
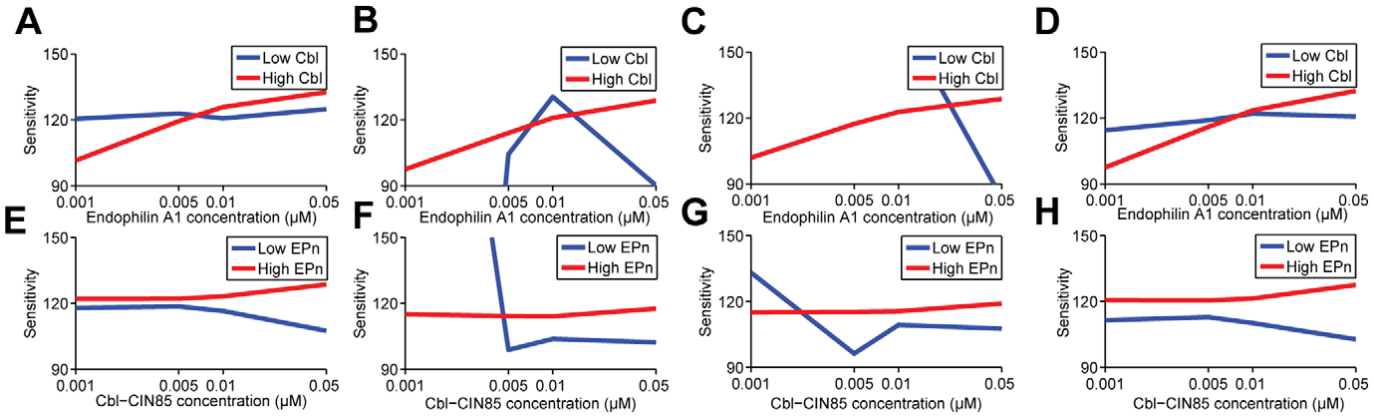
Detailed analysis on the sensitivities of EGFR endocytosis mediated by scaffold proteins KSR and MP1 coregulated by Cbl-CIN85 and Endophilin A1. Sensitivities of endocytosed EGFR toward EGF variation (A to D) regulated by Endophilin A1 concentration variation when Cbl-CIN85 is low (0.0001 µM) or high (0.8 µM); (E to H) Cbl-CIN85 concentration variation when Endophilin A1 is low (0.0001 µM) or high (0.8 µM) at four conditions: (A, E) when both scaffolds are present in suboptimal “low” levels [KSR  = 0.02 µM, MP1  = 0.02 µM]; (B, F) when both scaffolds are present in optimally “high” concentrations as determined earlier [KSR  = 0.3 µM, MP1  = 0.3 µM]; (C, G) when KSR is present at “high” level [0.3 µM] and MP1 at “low” level [0.02 µM]; (D, H) when MP1 is present at “high” level [0.3 µM] and KSR is at “low” level [0.02 µM].

Since Cbl-CIN85 and Endophilin A1 promote endocytosis of activated EGF receptors and facilitate trafficking of the signaling complex to late endosomes, we went on to analyze the concomitant modulation of receptor endocytosis in addition to the dynamics of ERK activity (**Figure 13**). Our analyses showed that, when the levels of scaffold proteins KSR and/or MP1 was either high, low or optimal, the sensitivity of endocytosed EGFR increased with increasing concentrations of Endophilin A1, if only when Cbl-CIN85 was present at high levels (**Figure 13**, **Panels A**, **B**, **C**, **D**). However, when the level of Cbl-CIN85 was low, the sensitivity underwent a dramatic phase change with a peak detected if KSR was present at high concentrations (**Figure 13**, **Panels B**, **C**) but not when both KSR and MP1 were present at suboptimal concentrations (**Figure 13** **, Panel A**) or when MP1 alone was present at high levels but KSR was at low levels (**Figure 13**, **Panel D**). In comparison, the sensitivity of endocytosed EGFR appeared to remain constant with increasing Cbl-CIN85 levels under high Endophilin A1 level (**Figure 13**, **Panels E**, **F**, **G**, **H**). However, when the Endophilin A1 level was low, this sensitivity underwent a dramatic decrease until it reached a stable range when KSR was at optimal concentration (**Figure 13**, **Panels F**, **G**). Interestingly, this dramatic decrease was abolished when both scaffold proteins were present at suboptimal concentrations (**Figure 13**, **Panel E**) or when only MP1 was present at a high level (**Figure 13**, **Panel H**). Under both conditions, the systems exhibited rather moderate change in the sensitivity of endocytosed EGFR toward varying EGF concentrations (**Figure 13**, **Panels E**, **H**). These results imply that at high concentrations, KSR could exert a more profound effect on the sensitivity of endocytosed EGFR (**Figure 13**, **Panels B**, **C**, **F**, **G**) probably by virtue of its ability to directly influence signaling cascades upstream of Ras/Erk signaling, thus affecting the impact more readily than those exerted by MP1.

By comparing both the sensitivity of endocytosed EGFR and the sensitivity of activating endosomal ERK together, our simulations further reveal that KSR and MP1 exert differential impacts on these two responses. In particular, it can be seen that changes to the ERK sensitivity appear to be more gradual and “analog-like” when compared to those for the endocytosed EGFR (which is more “digital-like”) when KSR was present in optimally high levels (**comparing** **Figures 12** **Panels F**, **G**, **N**, **O with** **Figures 13**, **Panels B**, **C**, **F**, **G**). Furthermore, in general, MP1 appears to maintain a more robust endosomal ERK activation (i.e. with lower sensitivity values <100; **Figure 12**) than the endocytosed EGFR (i.e. with sensitivity values >90; **Figure 13**). These results are therefore consistent with the view that KSR exerts less influence on the ligand sensitivity of the ERK endosomal subpathway (**see** **Figure 12**, **Panels E to H**, **and M to P**).

Taken together, our combined analyses on the dynamics of EGFR endocytosis and ERK activation define their unique responses that depend closely on the relative levels of not just the key scaffold proteins KSR and MP1, but also the influence by the endosomal regulators, Cbl-CIN85 and Endophilin A1. These analyses further support the multi-dimensional regulation of ligand sensitivity by the scaffold proteins and endocytosis regulators in a ligand-based Ras/Erk regulation.

### SHP2 can influence ligand sensitivities of ERK activation

EGF receptor signaling complex undergoes dynamic activation and feedback inhibition in order to ensure faithful propagation and integration. There is plenty of evidence supporting the positive role of the phosphotyrosine phosphatase SHP2 on the Ras/MAPK pathway and its association with receptor endocytosis; many of which are highly cell type- and stimuli-dependent [67], [68], [69]. For example, tyrosine phosphorylation of SHP2 is required for normal ERK activation in response to PDGF, but not by EGF or IGF in fibroblasts [68]. However, much less is known about how SHP2, as an important upstream signaling component, would contribute to the multi-dimensional ligand sensitivity regulation on ERK activation.

In order to examine this possible effect, we incorporated the double negative feedback loop (SHP2 –––| phosphorylated EGFR-RasGAP –––| RasGTP; where “–––|” denotes inhibition) to capture the possible main functions of SHP2 on ERK activation [70], [71], [72]. Our initial analyses showed that under the positive influence of SHP2 (**Supplementary Figure S6**), the sensitivities of ERK activation by the scaffolds, Cbl-CIN85 and Endophilin A1 coregulation undergoes dramatic fluctuations. For examples, the sensitivities of ERK activation from the membrane subpathway increased with increasing Endophilin A1 concentration when Cbl-CIN85 level was high, but in an opposite direction, implying that EGF causes negative effect on ERK activation (**Supplementary Figure S7**, **Panels A and C**). In contrast, the sensitivity from the endosomal subpathway remained positive and it appeared to be multi-phasic (more peaks observed) with increasing Cbl-CIN85 and Endophilin A1 (**Supplementary Figure S7**, **Panels B and D**). The current “all-or-none” EGFR phosphorylation model therefore provides some clues that SHP2 could indeed perturb the sensitivity of ERK activation. However, in the absence of more detailed information about the kinetics and activity of various phosphorylated species of the EGFR which SHP2 acts on, further analyses and comparison of the endocytosed EGFR with the dynamic ERK activation become somewhat limited, and would await further investigation.

### Concluding remarks

Sensitivities of pathways reflect the ability of a system to adjust itself to react against varying environments, and robustness against intracellular and extracellular perturbations. Our newly integrative simulation model, optimized and validated against a number of experimental and published simulation results, reveals the collective effects of KSR and MP1 on ERK activation and ligand sensitivity, depending on the relative levels of these scaffold proteins and also the immediate regulators. While being able to predict variations of ERK activation induced by KSR and MP1 knockout, our simulation also reveals that KSR synergistically enhances signaling via the conventional EGFR-Ras-Raf-MEK-ERK pathway. However, the effect from MP1 appears to be additive to that of KSR-mediated pathway and unlike KSR, it is insensitive towards EGF variations across the range of 25–100 ng/ml unless the level of EGF is present at much lower level and there is an reciprocal change in the levels of Endophilin A1 and Cbl-CIN85. Under such conditions, the inert response can be reversed by increasing levels of Endophilin A1 while keeping the levels of Cbl-CIN85 low (**Figure 12**, **Panels E**, **F**, **G**, **H**) or by increasing level of Cbl-CIN85 while keeping the level of Endophilin A1 low (**Figure 12**, **Panels M**, **N**, **O**, **P**). Further analyses on the ligand sensitivity of endocytosed EGFR showed that high levels of KSR exerts more profound effect on the response whereas MP1 helps maintain the robustness of the endosomal ERK activation instead of the endocytosed EGFR.

**Figure 14:**
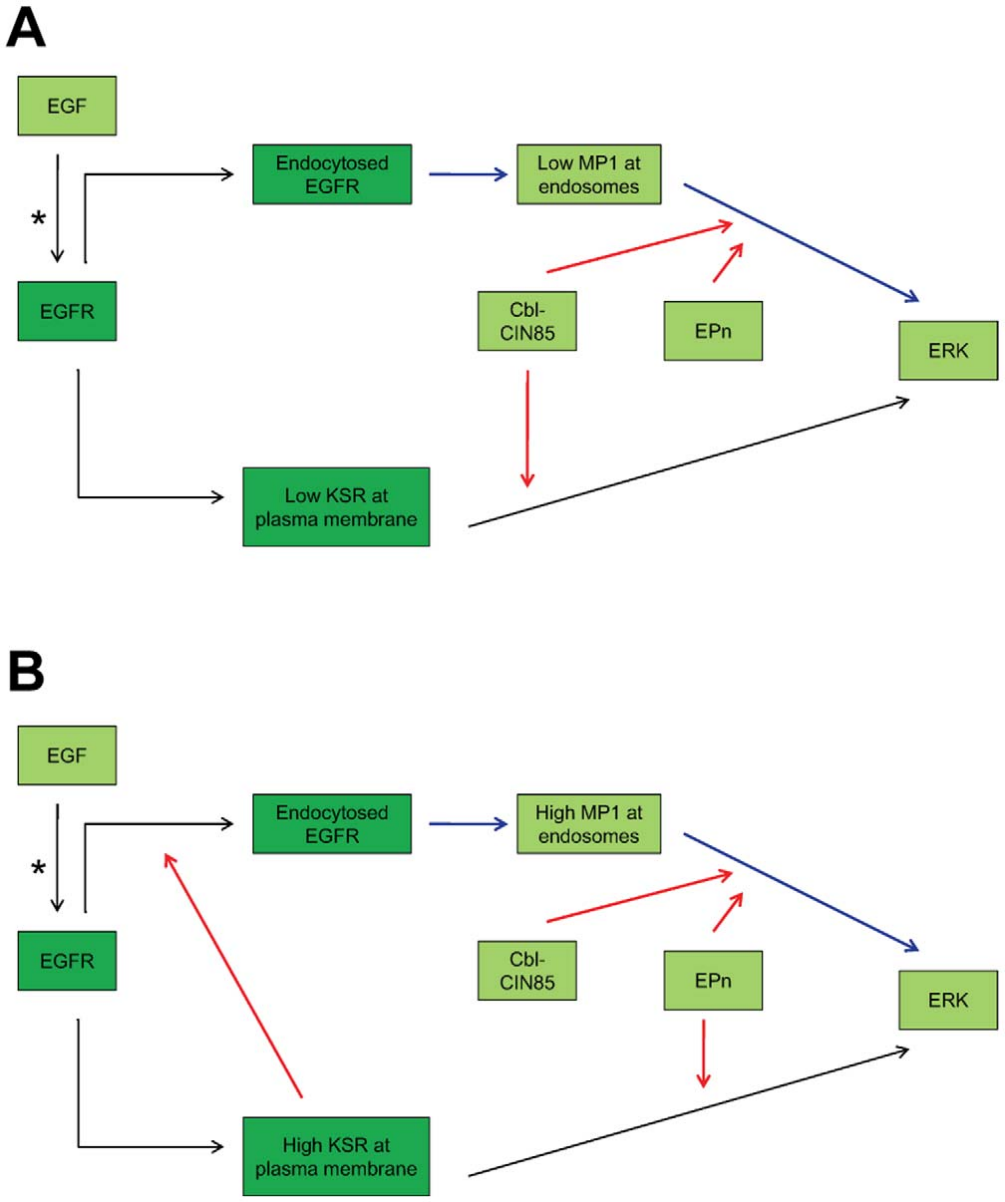
Distinct dynamics of EGFR endocytosis and ERK activation sensitivities mediated by scaffolds KSR and MP1, co-regulated by Cbl-CIN85 and Endophilin A1. EGF-induced ERK activation are collectively regulated at two different compartments (subpathways): near the plasma membrane (the conventional EGFR-Ras-Raf-MEK-ERK module and KSR-mediated signaling), and at late endosomes (MP1-mediated signaling). In this model, (**A**) when scaffold proteins are present at low levels, the two subpathways show distinctive sensitivities toward growth factor stimulation. MP1 exerts a more robust response to both endocytosed EGFR and ERK activation (lower sensitivity, denoted by blue lines) which could be influenced further by both Cbl-CIN85 and Endophilin (denoted by red lines). However, KSR has little effect on endocytosed EGFR but it affects sensitivity of ERK activation, co-regulated mainly by Cbl-CIN85 (denoted by red line) and not by Endophilin A1. (**B**) When both scaffold proteins are present at high levels, the sensitivity of endocytosed EGFR becomes milder and “analog-like” for the endosomal ERK activation (denoted by blue line) which is also co-regulated by both Cbl-CIN85 and Endophilin (denoted by red lines). However, high levels of KSR could now exert greater influence on the sensitivity of endocytosed EGFR (denoted by red line) to become more “digital” while the sensitivity of ERK mediated by KSR is further regulated more profoundly by Endophilin A1 (denoted by red line) but less by Cbl-CIN85. For further clarify, *light green boxes* denote components contributing towards lower sensitivity, *high green boxes* denote components contributing towards higher sensitivity.

In summary, using scaffold-mediated signaling as an example, we have demonstrated that various components in the EGF signaling pathway have distinct contributions (i.e. scaffolds or regulators) and they respond and act in concert to execute the final signal output as a function of varying EGF concentrations and times after stimulation (**Figure 14**). While the actual physiological significance of this multi-level and cross-regulation effects remain to be verified experimentally, the current model provides an attractive platform to further integrate the input of other scaffolds and regulators such as the Sef, Mek, GEFs and GAPs [32], [33], [35], [73] as well as a higher level of control via scaffold dimerization and the interactions among different scaffold proteins. These serve as a regulatory hub to fine-tune ERK signaling in response to different fluxes of physiological or pathophysiological stimuli. Understanding the intricate interplay and their differences in normal and pathophysiological conditions should help shed light to the possible mechanism of their involvement in cancer [24], [74], inflammation [22], [23], adipogenesis [19], cardiovascular disease [7], [8], urinary bladder dysfunction [9], and in the response to anti-proliferative agents targeting these proteins and pathways [75].

## Methods

### (a) Model construction and components

The pathway model used here is illustrated schematically in **Figure 1**. Two cascades were added to our earlier EGFR-ERK simulation model [35], [54]. These are the KSR and MP1 cascades based on the published models of the MAPK cascade with generic scaffold proteins [27], [28] as illustrated in **Figures 2** **and** **3**, respectively. These models were based on the following assumptions made by Levchenko et al [27] and supported by experimental data: (i) These scaffold proteins do not bind partially or fully activated kinases, based on the observation that MP1 has no effect on MEK-1 previously activated by B-Raf [61]. (ii) Kinase activation by a scaffold protein is processive rather than distributive, based on two observations that MP1 increases B-raf activation of MEK-1 and that dual phosphorylation of MAPK at two sites (necessary for MAPK activation) take place simultaneously in the presence of MEF (a MEK-enhancing factor from rabbit skeletal muscle) whereas phosphorylation at the second site is delayed by about 20 min in the absence of MEF [76]. (iii) The catalytic activity of a scaffold protein can be precluded from the model, as supported by the finding that the scaffolding function of KSR is independent of its kinase activity [77], [78]. (iv) Kinases bind to scaffold proteins independent of each other, as revealed by some experimental studies [61], [79]. (v) There is no inter-scaffold protein interaction, based on the fact that although p14 and MP1 were suggested to be able to weakly self-associate in vitro [80], there has been no reports about such homodimers being detected in experimental systems to date.

The constituent molecular interactions, their kinetic constants, and molecular concentrations are detailed in **Supplementary Tables S1 and S2**. The ordinary differential equations describing these interactions were derived based on mass action laws with interaction rate constants defined by the forward and reverse rate constants k_f_ and k_b_ or turnover k_cats_ value used in the published models [29], [31], [32], [33], [34], [81] or reported from other literature. Our simulation model contains 541 equations and interactions and 412 distinct molecular species, characterized by 755 kinetic parameters (with 238 unique parameters) and 59 initial molecular concentrations. The model is built using kroneckerbio - a matlab toolbox developed in Tidor's lab, MIT. A fourth order Runge-Kutta method with adaptive step-size control was used for integrating these equations. These ODEs were then solved using the Ode15 solver of Matlab. The reactions and initial conditions of our model are provided in the Supplementary Material (**Supplementary Text S1, Supplementary Tables S1 and S2**).

### (b) Collection and estimation of kinetic parameters

The types of parameters used in our model are protein-protein interactions and catalytic activities. The published simulation studies have shown that most parameters are robust and insensitive to significantly alter the overall pathway behavior [31], [33]. Apart from the use of the parameters of the published simulation models, additional parameters were obtained from the literature based on the widely used assumption that the parameters measured in vitro and in some cell lines are generally applicable in most cases. For those protein-protein interactions with unavailable parameters, their parameters were estimated from the known parameters of the relevant interacting domain profile pairs [82], [83] or other interacting protein pairs of similar sequences.

### (c) Sensitivity analysis

To quantify how ERK activation in these two different compartments responds to changing EGF concentrations, the total amount of active ERK produced in the first 1000 seconds as the objective function, and full derivatives of the objective function 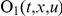 with respect to initial conditions of EGF is used to calculate the sensitivity.
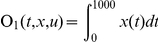
and sensitivity is computed as:
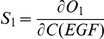



Similarly, the ligand sensitivity on endocytosized EGFR is quantified by calculating the derivatives of the total amount of endocytosized EGFR (denoted as “EGF-pEGFR-2-Grb2-SOS_e” in **reactions 158 and 159, Supplementary Table S1**) in the first 1000 seconds towards EGF variation.
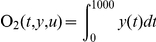
and sensitivity is computed as:
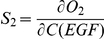



Since c-Cbl-CIN85 and Endophilin A1 will affect the duration of ERK activation through regulation of EGFR degradation and turnover, we want to know whether and to what extent c-Cbl-mediated endocytosis pathway affect the ERK activation sensitivities in the two different compartments toward variation of EGF stimulation.

To this end, we calculated the effect of c-Cbl-CIN85 and Endophilin A1 concentration on the ERK production sensitivity from the two sub-pathways (membrane and endosomal) toward EGF concentration, respectively and in a combinatorial manner. 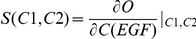



Here, C1 is the initial concentration of c-Cbl-CIN85, C2 is the initial concentration of Endophilin A1.

### (d) Effect of scaffold concentrations on sensitivity analysis

To evaluate the effect of scaffold protein concentrations on Cbl and Endophilin A1-mediated compartment-specific response toward EGF variation, we set the level of KSR and MP1 to the ones optimal for signaling (which is the concentration for the maximum total amount of active ERK produced in the first 1000 seconds across 0 – 1.0 µM scaffold concentration, **Figure 4B** **and** **6B**), and calculate the sensitivities under different conditions again.

## Supporting Information

Figure S1 Simulated profile of active ERK stimulated by 100 ng/ml EGF, consistent with the observation that treatment of 100 ng/ml EGF in PC12 cells transiently activates ERK, which peaks within 5 minutes and decays within 30–60 minutes [55].
(TIF)

Figure S2 Simulated profile of activated Ras stimulated by 100 ng/ml EGF, consistent with the observation that active RasGTP levels in EGF-treated PC12 cells increase dramatically within 5 minutes and decay steeply within 10 minutes [33].
(TIF)

Figure S3 Profile of active ERK at different PP2A concentrations, consistent with another simulation work by Mayawala et al. [62].
(TIF)

Figure S4 Profile of active ERK at different MKP3 concentrations, consistent with another simulation work by Mayawala et al. [62].
(TIF)

Figure S5 The relative sensitivity of ppERK from these two subpathways for EGF, 1. membrane subpathway (KSR-mediated and conventional one) 2. endosomal subpathway (MP1-mediated one).
(TIF)

Figure S6 SHP2 knockout simulation, consistent with experimental result that in PC12 cells, the expression of a dominant negative mutant of SHP2 (SHP2-C/S) only causes a minor reduction of the pERK levels [67].
(TIF)

Figure S7 Differential sensitivity of ppERK in “SHP2 positive model” from membrane (A, C) and endosomal (B, D) subpathways under various Cbl-CIN85 and Endophilin A1 concentrations when: (A, B) both scaffolds KSR and MP1 are at suboptimal level; (C, D) both scaffold proteins are at optimal level.
(TIF)

Table S1 List of chemical reactions and related kinetic parameters used in the model. The relevant references from which the parameters obtained are given in PubMed ID. Some of the kinetic values used in this study are not necessary exactly the same as the values given in the cited references but were scaled and optimized in 10-fold ranges according to the performance and kinetics of current model. For those kinetic parameters that are not readily available, parameter values from their homologs partners were taken and were subsequently scaled and optimized in 10-fold ranges (denoted as “Estimated” in the Table).
(DOC)

Table S2 List of species and initial concentrations used in the model.
(DOC)

Text S1 A detailed description of the signaling model used in this study.
(DOC)
