# Supplementary figures and images for: Simulating EGFR-ERK Signaling Control by Scaffold Proteins KSR and MP1 Reveals Differential Ligand-Sensitivity Co-Regulated by Cbl-CIN85 and Endophilin
Source: PLoS One. 2011 Aug 1;6(8):e22933. doi: 10.1371/journal.pone.0022933 (PMC3148240; doi:10.1371/journal.pone.0022933)

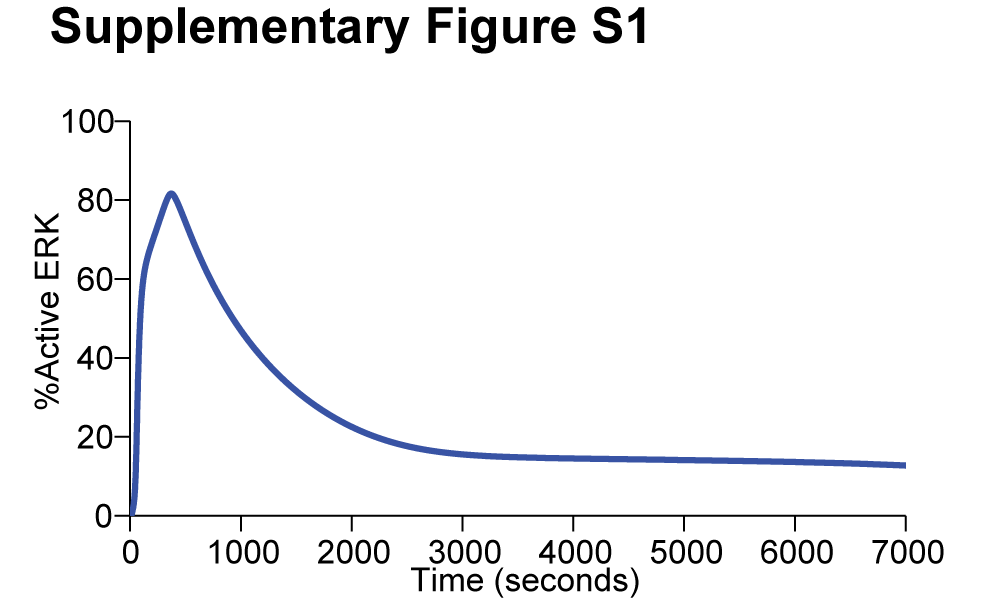

Supplement: Figure S1 — Simulated profile of active ERK stimulated by 100 ng/ml EGF, consistent with the observation that treatment of 100 ng/ml EGF in PC12 cells transiently activates ERK, which peaks within 5 minutes and decays within 30–60 minutes [55]. (TIF) [file pone.0022933.s001.tif]

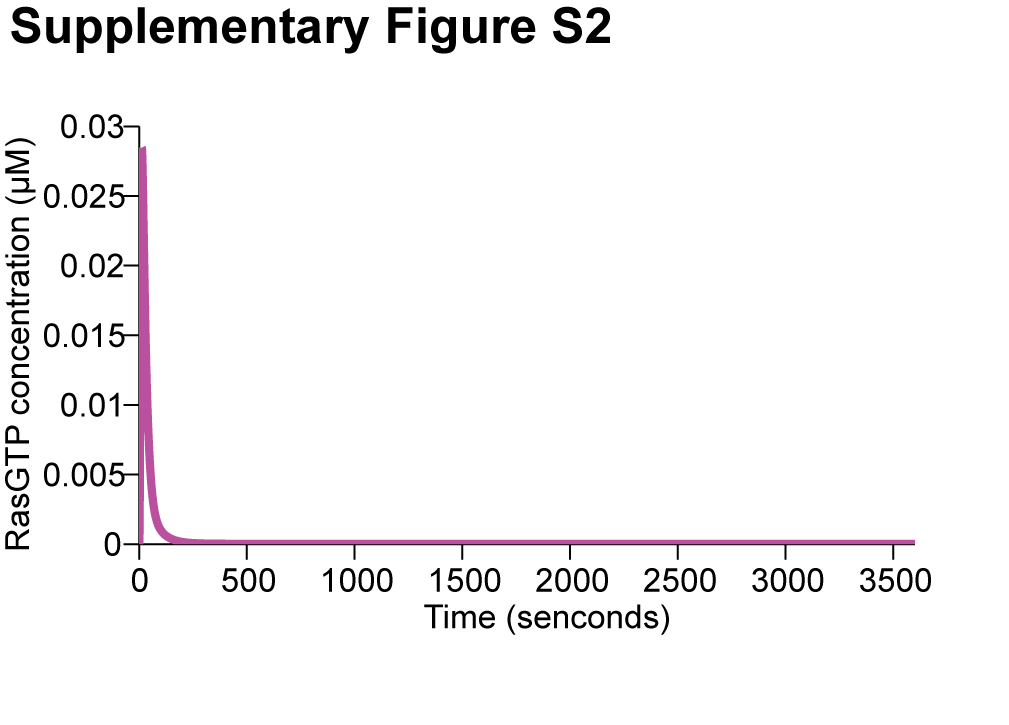

Supplement: Figure S2 — Simulated profile of activated Ras stimulated by 100 ng/ml EGF, consistent with the observation that active RasGTP levels in EGF-treated PC12 cells increase dramatically within 5 minutes and decay steeply within 10 minutes [33]. (TIF) [file pone.0022933.s002.tif]

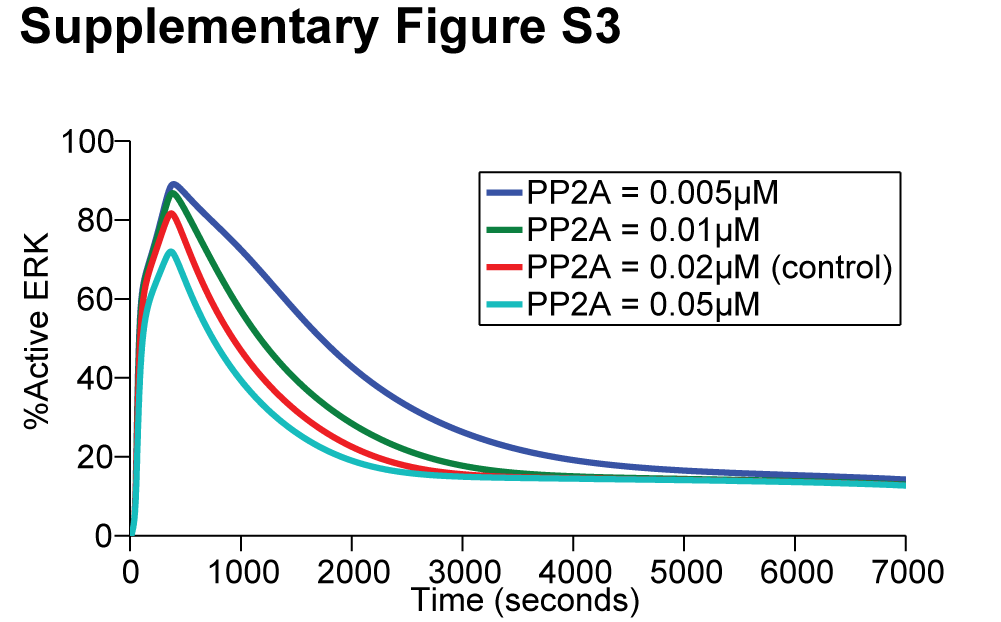

Supplement: Figure S3 — Profile of active ERK at different PP2A concentrations, consistent with another simulation work by Mayawala et al. [62]. (TIF) [file pone.0022933.s003.tif]

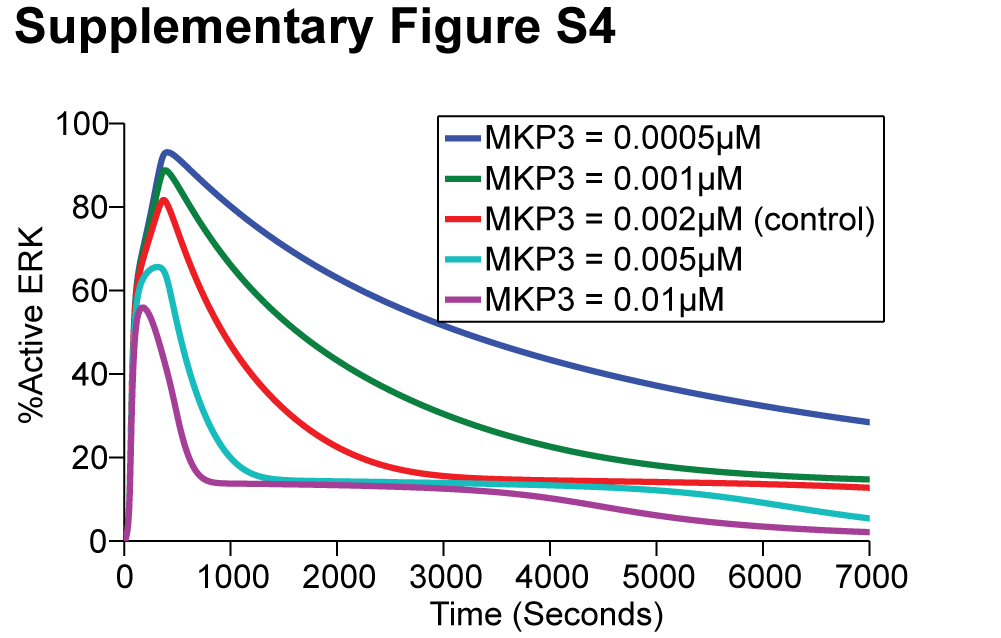

Supplement: Figure S4 — Profile of active ERK at different MKP3 concentrations, consistent with another simulation work by Mayawala et al. [62]. (TIF) [file pone.0022933.s004.tif]

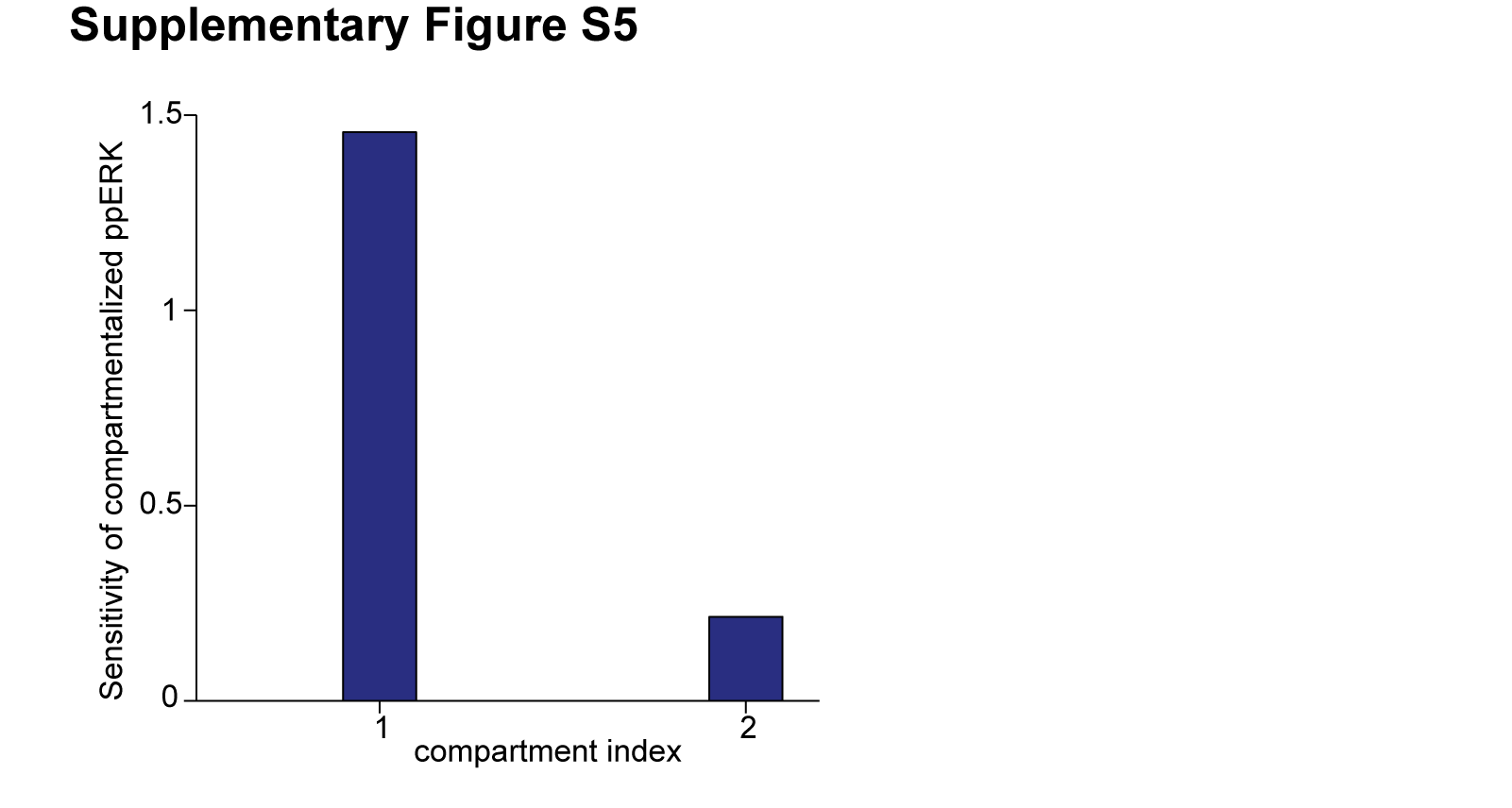

Supplement: Figure S5 — The relative sensitivity of ppERK from these two subpathways for EGF, 1. membrane subpathway (KSR-mediated and conventional one) 2. endosomal subpathway (MP1-mediated one). (TIF) [file pone.0022933.s005.tif]

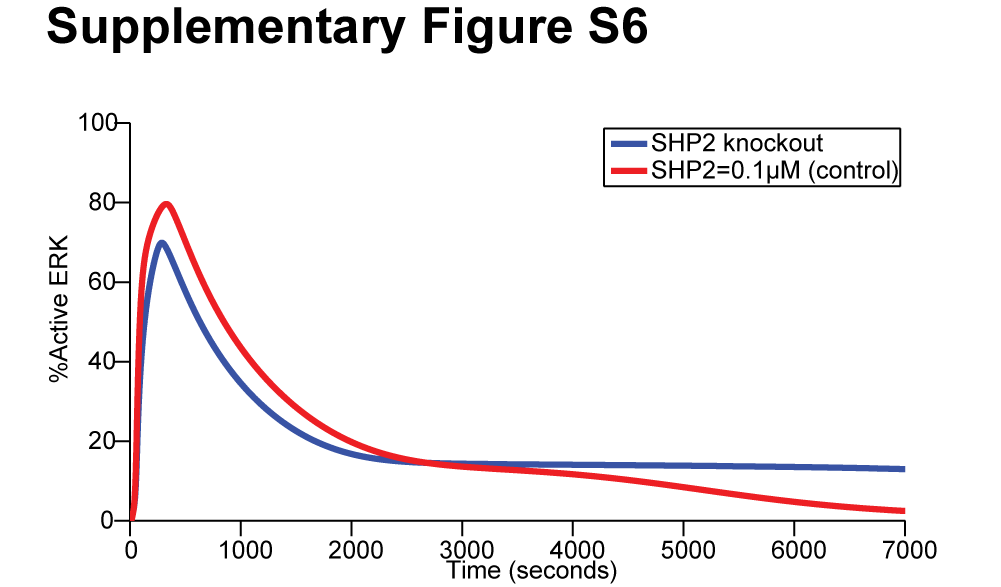

Supplement: Figure S6 — SHP2 knockout simulation, consistent with experimental result that in PC12 cells, the expression of a dominant negative mutant of SHP2 (SHP2-C/S) only causes a minor reduction of the pERK levels [67]. (TIF) [file pone.0022933.s006.tif]

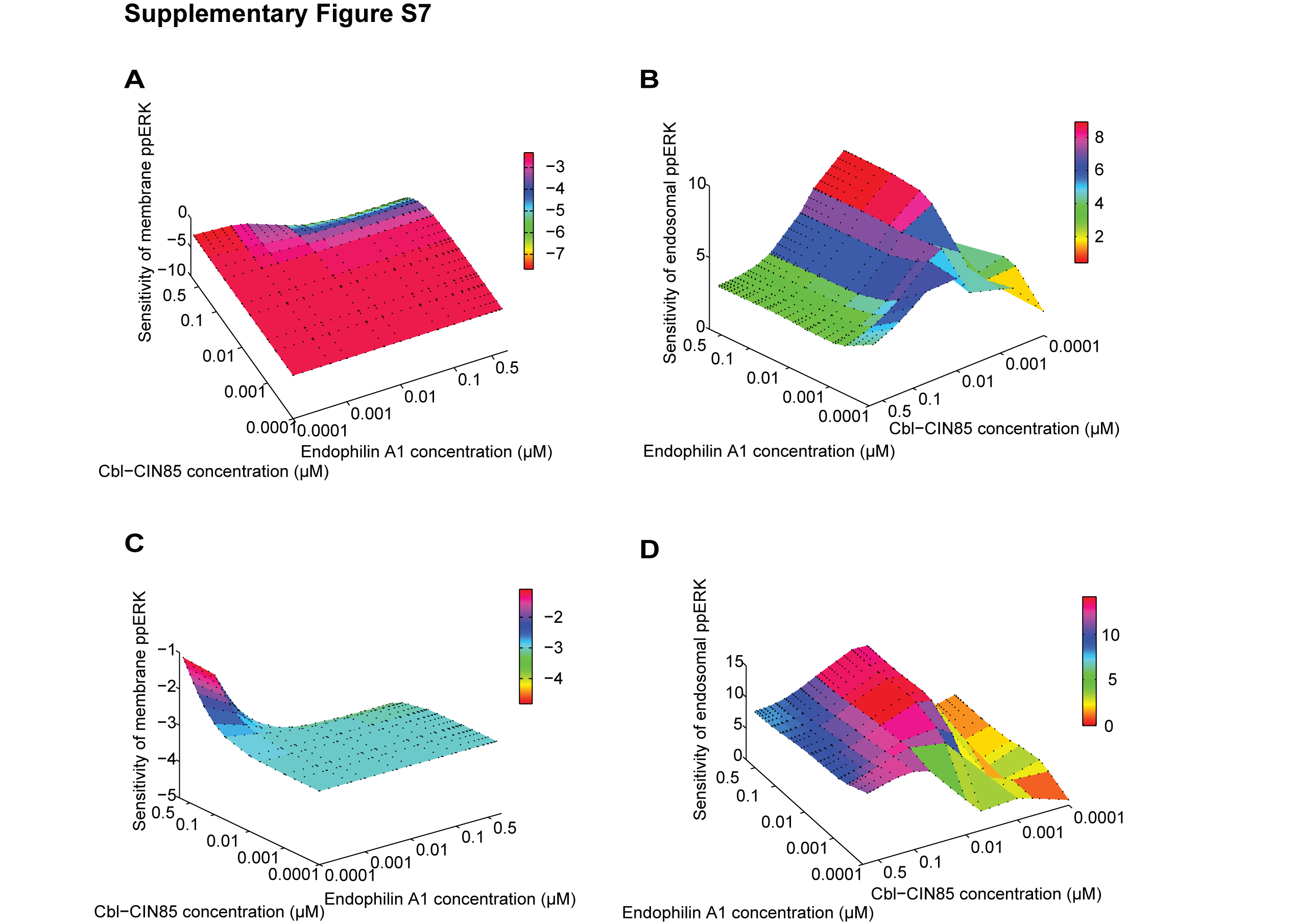

Supplement: Figure S7 — Differential sensitivity of ppERK in “SHP2 positive model” from membrane (A, C) and endosomal (B, D) subpathways under various Cbl-CIN85 and Endophilin A1 concentrations when: (A, B) both scaffolds KSR and MP1 are at suboptimal level; (C, D) both scaffold proteins are at optimal level. (TIF) [file pone.0022933.s007.tif]
