# Supplementary material for: Simulating EGFR-ERK Signaling Control by Scaffold Proteins KSR and MP1 Reveals Differential Ligand-Sensitivity Co-Regulated by Cbl-CIN85 and Endophilin
Source: PLoS One. 2011 Aug 1;6(8):e22933. doi: 10.1371/journal.pone.0022933 (PMC3148240; doi:10.1371/journal.pone.0022933)
Supplement: Table S1 — List of chemical reactions and related kinetic parameters used in the model. The relevant references from which the parameters obtained are given in PubMed ID. Some of the kinetic values used in this study are not necessary exactly the same as the values given in the cited references but were scaled and optimized in 10-fold ranges according to the performance and kinetics of current model. For those kinetic parameters that are not readily available, parameter values from their homologs partners were taken and were subsequently scaled and optimized in 10-fold ranges (denoted as “Estimated” in the Table). (DOC) [file pone.0022933.s008.doc]

**Supplementary Table S1.** List of chemical reactions and related kinetic parameters used in the model. The relevant references from which the parameters obtained are given in PubMed ID. Some of the kinetic values used in this study are not necessary exactly the same as the values given in the cited references but were scaled and optimized in 10-fold ranges according to the performance and kinetics of current model. For those kinetic parameters that are not readily available, parameter values from their homologs partners were taken and were subsequently scaled and optimized in 10-fold ranges (denoted as “Estimated” in the Table).

| **Reaction Number** | **Chemical Reactions and Description** | **k_f_ (uM.s)^-1^** | **k_b_ (s)^-1^** | **K_cat_ (s)^-1^** | **References (PMID)** |
| --- | --- | --- | --- | --- | --- |
|  | ***Activation and inactivation from EGFR to Ras at plasma membrane*** |  |  |  |  |
| **1** | EGF + EGFR = EGF-EGFR | 100 | 0.0038 |  | 14751248; 11923843 |
| **2** | EGF-EGFR + EGF-EGFR = EGF-EGFR-2 | 10 | 0.02 |  | 14751248; 15793571 |
| **3** | EGF-EGFR-2 -> EGF-pEGFR-2 |  |  | 2.014 | 14751248 |
| **4** | EGF-pEGFR-2 + SHP = EGF-pEGFR-2-SHP | 3.14 | 0.2 |  | 14751248 |
| **5** | EGF-pEGFR-2-SHP -> EGF-EGFR-2 + SHP |  |  | 0.2661 | 14751248 |
| **6** | EGF-pEGFR-2 + Shc = EGF-pEGFR-2-Shc | 90 | 0.6 |  | 10514507; 14751248 |
| **7** | EGF-pEGFR-2-Shc -> EGF-pEGFR-2-pShc |  |  | 0.5838 | 14751248 |
| **8** | EGF-pEGFR-2-pShc = EGF-pEGFR-2 + pShc | 0.3 | 4.481 |  | 14751248 |
| **9** | pShc + SHP = pShc-SHP | 3.114 | 0.2 |  | 14751248 |
| **10** | pShc-SHP -> Shc + SHP |  |  | 0.2661 | 14751248 |
| **11** | EGF-pEGFR-2-pShc + Grb2 = EGF-pEGFR-2-pShc-Grb2 | 3 | 0.1 |  | 10514507 |
| **12** | EGF-pEGFR-2-pShc-Grb2 + SOS = EGF-pEGFR-2-pShc-Grb2-SOS | 10 | 0.0214 |  | 10514507 |
| **13** | Grb2 + SOS = Grb2-SOS | 0.1 | 0.0015 |  | 10514507 |
| **14** | EGF-pEGFR-2-pShc + Grb2-SOS = EGF-pEGFR-2-pShc-Grb2-SOS | 10 | 0.045 |  | 15793571; 14571248 |
| **15** | EGF-pEGFR-2-pShc-Grb2-SOS + RasGDP = EGF-pEGFR-2-pShc-Grb2-SOS-RasGDP | 202.9 | 0.18 |  | 14751248 |
| **16** | EGF-pEGFR-2-pShc-Grb2-SOS-RasGDP -> EGF-pEGFR-2-pShc-Grb2-SOS + RasGTP |  |  | 40 | 14751248 |
| **17** | EGF-pEGFR-2 + Grb2 = EGF-pEGFR-2-Grb2 | 3 | 0.05 |  | 10514507 |
| **18** | EGF-pEGFR-2-Grb2 + SOS = EGF-pEGFR-2-Grb2-SOS | 10 | 0.06 |  | 10514507 |
| **19** | EGF-pEGFR-2 + Grb2-SOS = EGF-pEGFR-2-Grb2-SOS | 2.734 | 0.025 |  | 14751248 |
| **20** | EGF-pEGFR-2-Grb2-SOS + RasGDP = EGF-pEGFR-2-Grb2-SOS-RasGDP | 202.9 | 0.18 |  | 14751248 |
| **21** | EGF-pEGFR-2-Grb2-SOS-RasGDP -> EGF-pEGFR-2-Grb2-SOS + RasGTP |  |  | 0.1434 | 14751248 |
|  | ***Conventional Raf-MEK-ERK signaling in the cytosol (scaffold-independent)*** |  |  |  |  |
| **22** | Raf + RasGTP = Raf-RasGTP | 1.754 | 0.05 |  | 14751248 |
| **23** | Raf-RasGTP -> pRaf + RasGTP |  |  | 0.7624 | 14751248 |
| **24** | pRaf + MEK1 = pRaf-MEK1 | 5.5 | 0.01833 |  | 11923843 |
| **25** | pRaf-MEK1 -> pRaf + pMEK1 |  |  | 3.5 | 11923843 |
| **26** | pRaf + pMEK1 = pRaf-pMEK1 | 5.5 | 0.01833 |  | 11923843 |
| **27** | pRaf-pMEK1 -> pRaf + ppMEK1 |  |  | 2.9 | 11923843 |
| **28** | ppMEK1 + ERK1 = ppMEK1-ERK1 | 3 | 0.033 |  | 14751248; 11923843 |
| **29** | ppMEK1-ERK1 -> ppMEK1 + pERK1 |  |  | 16 | 11923843 |
| **30** | ppMEK1 + pERK1 = ppMEK1-pERK1 | 3 | 0.033 |  | 14751248; 11923843 |
| **31** | ppMEK1-pERK1 -> ppMEK1 + ppERK1 |  |  | 5.7 | 11923843 |
| **32** | pRaf + MEK2 = pRaf-MEK2 | 5.5 | 0.01833 |  | 11923843 |
| **33** | pRaf-MEK2 -> pRaf + pMEK2 |  |  | 3.5 | 11923843 |
| **34** | pRaf + pMEK2 = pRaf-pMEK2 | 5.5 | 0.01833 |  | 11923843 |
| **35** | pRaf-pMEK2 -> pRaf + ppMEK2 |  |  | 2.9 | 11923843 |
| **36** | ppMEK1 + ERK2 = ppMEK1-ERK2 | 3 | 0.033 |  | 14751248; 11923843 |
| **37** | ppMEK1-ERK2 -> ppMEK1 + pERK2 |  |  | 16 | 11923843 |
| **38** | ppMEK1 + pERK2 = ppMEK1-pERK2 | 3 | 0.033 |  | 14751248; 11923843 |
| **39** | ppMEK1-pERK2 -> ppMEK1 + ppERK2 |  |  | 5.7 | 11923843 |
| **40** | ppMEK2 + ERK1 = ppMEK2-ERK1 | 3 | 0.033 |  | 14751248; 11923843 |
| **41** | ppMEK2-ERK1 -> ppMEK2 + pERK1 |  |  | 16 | 11923843 |
| **42** | ppMEK2 + pERK1 = ppMEK2-pERK1 | 3 | 0.033 |  | 14751248; 11923843 |
| **43** | ppMEK2-pERK1 -> ppMEK2 + ppERK1 |  |  | 5.7 | 11923843 |
| **44** | ppMEK2 + ERK2 = ppMEK2-ERK2 | 3 | 0.033 |  | 14751248; 11923843 |
| **45** | ppMEK2-ERK2 -> ppMEK2 + pERK2 |  |  | 16 | 11923843 |
| **46** | ppMEK2 + pERK2 = ppMEK2-pERK2 | 3 | 0.033 |  | 14751248; 11923843 |
| **47** | ppMEK2-pERK2 -> ppMEK2 + ppERK2 |  |  | 5.7 | 11923843 |
| **48** | pRaf + Pase = pRaf-Pase | 71.7 | 0.2 |  | 11923843 |
| **49** | pRaf-Pase -> Raf + Pase |  |  | 1 | 11923843 |
| **50** | ppMEK1 + PP2A = ppMEK-PP2A | 14.3 | 0.8 |  | 11923843 |
| **51** | ppMEK1-PP2A -> pMEK1 + PP2A |  |  | 0.058 | 11923843 |
| **52** | pMEK1 + PP2A = pMEK1-PP2A | 0.25 | 0.5 |  | 11923843 |
| **53** | pMEK1-PP2A -> MEK1 + PP2A |  |  | 0.058 | 11923843 |
| **54** | ppERK1 + MKP3 = ppERK1-MKP3 | 7 | 0.6 |  | 11923843 |
| **55** | ppERK1-MKP3 -> pERK1 + MKP3 |  |  | 0.27 | 11923843 |
| **56** | pERK1 + MKP3 = pERK1-MKP3 | 5 | 0.5 |  | 11923843 |
| **57** | pERK1-MKP3 -> ERK1 + MKP3 |  |  | 0.3 | 11923843 |
| **58** | ppERK1_k + MKP3 = ppERK1_k-MKP3 | 7 | 0.6 |  | 11923843 |
| **59** | ppERK1_k-MKP3 -> pERK1 + MKP3 |  |  | 0.27 | 11923843 |
| **60** | ppMEK2 + PP2A = ppMEK-PP2A | 14.3 | 0.8 |  | 11923843 |
| **61** | ppMEK2-PP2A -> pMEK2 + PP2A |  |  | 0.058 | 11923843 |
| **62** | pMEK2 + PP2A = pMEK2-PP2A | 0.25 | 0.5 |  | 11923843 |
| **63** | pMEK2-PP2A -> MEK2 + PP2A |  |  | 0.058 | 11923843 |
| **64** | ppERK2 + MKP3 = ppERK2-MKP3 | 7 | 0.6 |  | 11923843 |
| **65** | ppERK2-MKP3 -> pERK2 + MKP3 |  |  | 0.27 | 11923843 |
| **66** | pERK2 + MKP3 = pERK2-MKP3 | 5 | 0.5 |  | 11923843 |
| **67** | pERK2-MKP3 -> ERK2 + MKP3 |  |  | 0.3 | 11923843 |
| **68** | ppERK2_k + MKP3 = ppERK2_k-MKP3 | 7 | 0.6 |  | 11923843 |
| **69** | ppERK2_k-MKP3 -> pERK2 + MKP3 |  |  | 0.27 | 11923843 |
| **70** | RasGTP -> RasGDP |  |  | 1.667E-05 | 14751248 |
| **71** | RasGTP + RasGAP = RasGTP-RasGAP | 2.854 | 0.96 |  | 14751248 |
| **72** | RasGTP-RasGAP -> RasGDP + RasGAP |  |  | 7.6 | 14751248 |
| **73** | ppERK1 + EGF-pEGFR-2-pShc-Grb2-SOS = ppERK1-EGF-pEGFR-2-pShc-Grb2-SOS | 8.898 | 0.1 |  | 14751248 |
| **74** | ppERK2 + EGF-pEGFR-2-pShc-Grb2-SOS = ppERK2-EGF-pEGFR-2-pShc-Grb2-SOS | 8.898 | 0.1 |  | 14751248 |
| **75** | ppERK1-EGF-pEGFR-2-pShc-Grb2-SOS -> ppERK1 + EGF-pEGFR-2 + pShc + Grb2 + pSOS |  |  | 0.426 | 14751248 |
| **76** | ppERK2-EGF-pEGFR-2-pShc-Grb2-SOS -> ppERK2 + EGF-pEGFR-2 + pShc + Grb2 + pSOS |  |  | 0.426 | 14751248 |
| **77** | ppERK1_k + EGF-pEGFR-2-pShc-Grb2-SOS = ppERK1_k-EGF-pEGFR-2-pShc-Grb2-SOS | 8.898 | 0.1 |  | 14751248 |
| **78** | ppERK2_k + EGF-pEGFR-2-pShc-Grb2-SOS = ppERK2_k-EGF-pEGFR-2-pShc-Grb2-SOS | 8.898 | 0.1 |  | 14751248 |
| **79** | ppERK1_k-EGF-pEGFR-2-pShc-Grb2-SOS -> ppERK1_k + EGF-pEGFR-2 + pShc + Grb2 + pSOS |  |  | 0.426 | 14751248 |
| **80** | ppERK2_k-EGF-pEGFR-2-pShc-Grb2-SOS -> ppERK2_k + EGF-pEGFR-2 + pShc + Grb2 + pSOS |  |  | 0.426 | 14751248 |
| **81** | ppERK1 + EGF-pEGFR-2-Grb2-SOS = ppERK1-EGF-pEGFR-2-Grb2-SOS | 8.898 | 0.1 |  | 14751248 |
| **82** | ppERK2 + EGF-pEGFR-2-Grb2-SOS = ppERK2-EGF-pEGFR-2-Grb2-SOS | 8.898 | 0.1 |  | 14751248 |
| **83** | ppERK1-EGF-pEGFR-2-Grb2-SOS -> ppERK1 + EGF-pEGFR-2 + Grb2 + pSOS |  |  | 0.426 | 14751248 |
| **84** | ppERK2-EGF-pEGFR-2-Grb2-SOS -> ppERK2 + EGF-pEGFR-2 + Grb2 + pSOS |  |  | 0.426 | 14751248 |
| **85** | ppERK1_k + EGF-pEGFR-2-Grb2-SOS = ppERK1_k-EGF-pEGFR-2-Grb2-SOS | 8.898 | 0.1 |  | 14751248 |
| **86** | ppERK2_k + EGF-pEGFR-2-Grb2-SOS = ppERK2_k-EGF-pEGFR-2-Grb2-SOS | 8.898 | 0.1 |  | 14751248 |
| **87** | ppERK1_k-EGF-pEGFR-2-Grb2-SOS -> ppERK1_k + EGF-pEGFR-2 + Grb2 + pSOS |  |  | 0.426 | 14751248 |
| **88** | ppERK2_k-EGF-pEGFR-2-Grb2-SOS -> ppERK2_k + EGF-pEGFR-2 + Grb2 + pSOS |  |  | 0.426 | 14751248 |
| **89** | pSOS -> SOS |  |  | 0.002 | 15793571 |
|  | ***Activation and Inactivation of PI3K-Akt Component*** |  |  |  |  |
| **90** | EGF-pEGFR-2 + PI3K = EGF-pEGFR-2-PI3K | 14 | 0.1743 |  | 16687399 |
| **91** | EGF-pEGFR-2-PI3K -> EGF-pEGFF-2 + pPI3K |  |  | 33.72 | 16687399 |
| **92** | pPI3K + TP4 = pPI3K-TP4 | 1 | 0.038 |  | 16687399 |
| **93** | pPI3K-TP4 -> PI3K + TP4 |  |  | 0.595 | 16687399 |
| **94** | pPI3K + PIP2 = pPI3K-PIP2 | 25 | 3.5 |  | 16687399 |
| **95** | pPI3K-PIP2 -> pPI3K + PIP3 |  |  | 25 | 16687399 |
| **96** | Akt + PIP3 = Akt-PIP3 | 3 | 1 |  | 16687399 |
| **97** | Akt-PIP3 + PDK1 = Akt-PIP3-PDK1 | 3 | 1 |  | 16687399 |
| **98** | Akt-PIP3-PDK1 -> pAkt-PIP3 + PDK1 |  |  | 3 | 16687399 |
| **99** | pAkt-PIP3 = pAkt + PIP3 | 10 | 0.001 |  | 16687399 |
| **100** | pAkt-PIP3 + Takt = pAkt-PIP3-Takt | 1 | 0.9 |  | 16687399 |
| **101** | pAkt-PIP3-Takt -> Akt-PIP3 + Takt |  |  | 0.001 | 16687399 |
| **102** | pRaf + pAkt-PIP3 = pRaf-pAkt-PIP3 | 3 | 0.5 |  | 16687399 |
| **103** | pRaf-pAkt-PIP3 -> ppRaf + pAkt-PIP3 |  |  | 3 | 16687399 |
| **104** | ppRaf -> pRaf |  |  | 0.001 | 16687399 |
|  | ***Activation and inactivation cycle of RhoA*** |  |  |  |  |
| **105** | pROCK + PTEN = pROCK-PTEN | 1.1 | 0.033 |  | 16687399 |
| **106** | pROCK-PTEN -> pROCK + pPTEN |  |  | 16 | 16687399 |
| **107** | pPTEN + PIP3 = pPTEN-PIP3 | 5 | 0.5 |  | 16687399 |
| **108** | pPTEN-PIP3 -> pPTEN + PIP2 |  |  | 5 | 16687399 |
| **109** | pPTEN -> PTEN |  |  | 0.1298 | 16687399 |
| **110** | PIP3 -> PIP2 |  |  | 17 | 16687399 |
| **111** | PIP3 + RacGEF = PIP3-RacGEF | 10 | 0.0214 |  | 12829242 |
| **112** | PIP3-RacGEF + RacGDP = PIP3-RacGEFRacGDP | 2.029 | 0.18 |  | 12829242 |
| **113** | PIP3-RacGEF-RacGDP -> PIP3-RacGEF + RacGTP |  |  | 0.1434 | 12829242 |
| **114** | RhoGDI + RacGDP = RhoGDI-RacGDP | 2.845 | 0.96 |  | Estimated |
| **115** | RacGTP -> RacGDP |  |  | 0.262 | 9535855 |
| **116** | RacGTP + RacGAP = RacGTP-RacGAP | 2.845 | 0.96 |  | 9535855 |
| **117** | RacGTP-RacGAP -> RacGDP + RacGAP |  |  | 1.205 | 9535855 |
| **118** | RhoGDP + RhoGDI = RhoGDP-RhoGDI | 20.29 | 0.18 |  | Estimated |
| **119** | RhoGDP + pRhoGEF = RhoGDP-pRhoGEF | 20.29 | 0.18 |  | 12423633 |
| **120** | RhoGDP-pRhoGEF -> RhoGTP + pRhoGEF |  |  | 4.98 | 12423633 |
| **121** | RhoGTP -> RhoGDP |  |  | 0.262 | 12423633 |
| **122** | EGF-pEGFR-2 + RasGAP = EGF-pEGFR-2-RasGAP | 0.1 | 0.01 |  | 14751248 |
| **123** | EGF-pEGFR-2-RasGAP + RasGTP = EGF-pEGFR-2-RasGAP-RasGTP | 2.854 | 0.96 |  | 14751248 |
| **124** | EGF-pEGFR-2-RasGAP-RasGTP -> EGF-pEGFR2-RasGAP + RasGDP |  |  | 7.76 | 14751248 |
| **125** | EGF-pEGFR-2-pShc-Grb2 + SHP2 = EGF-pEGFR-2-pShc-Grb2-SHP2 | 10 | 1 |  | 14751248 |
| **126** | EGF-pEGFR-2-Grb2 + SHP2 = EGF-pEGFR-2-Grb2-SHP2 | 10 | 1 |  | 14751248 |
| **127** | EGF-pEGFR-2-pShc-Grb2-SHP2 -> EGF-EGFR-2 + pShc + Grb2 + SHP2 |  |  | 2.661 | 14751248 |
| **128** | EGF-pEGFR-2-Grb2-SHP2 -> EGF-EGFR-2 + Grb2 + SHP2 |  |  | 2.661 | 14751248 |
| **129** | EGF-pEGFR-2-pShc-Grb2-SHP2 + pRhoGEF = EGF-pEGFR-2-pShc-Grb2-SHP2-pRhoGEF | 3.114 | 0.2 |  | 14751248 |
| **130** | EGF-pEGFR-2-pShc-Grb2-SHP2-pRhoGEF -> EGF-pEGFR-2-pShc-Grb2-SHP2 + RhoGEF |  |  | 2.661 | 14751248 |
| **131** | EGF-pEGFR-2-pShc-Grb2-SHP2 + pRhoGAP = EGF-pEGFR-2-pShc-Grb2-SHP2-pRhoGAP | 3.114 | 0.2 |  | 14751248 |
| **132** | EGF-pEGFR-2-pShc-Grb2-SHP2-pRhoGAP -> EGF-pEGFR-2-pShc-Grb2-SHP2 + RhoGAP |  |  | 2.661 | 14751248 |
| **133** | EGF-pEGFR-2-Grb2-SHP2 + pRhoGEF = EGF-pEGFR-2-Grb2-SHP2-pRhoGEF | 3.114 | 0.2 |  | 14751248 |
| **134** | EGF-pEGFR-2-Grb2-SHP2-pRhoGEF -> EGF-pEGFR-2-Grb2-SHP2 + RhoGEF |  |  | 2.661 | 14751248 |
| **135** | EGF-pEGFR-2-Grb2-SHP2 + pRhoGAP = EGF-pEGFR-2-Grb2-SHP2-pRhoGAP | 3.114 | 0.2 |  | 14751248 |
| **136** | EGF-pEGFR-2-Grb2-SHP2-pRhoGAP -> EGF-pEGFR-2-Grb2-SHP2 + RhoGAP |  |  | 2.661 | 14751248 |
| **137** | EGF-pEGFR-2-RasGAP + SHP2 = EGF-pEGFR-2-RasGAP-SHP2 | 3.114 | 0.2 |  | 14751248 |
| **138** | EGF-pEGFR-2-RasGAP-SHP2 -> EGF-EGFR-2 + RasGAP + SHP2 |  |  | 2.661 | 14751248 |
| **139** | pSrc + RhoGEF = pSrc-RhoGEF | 40 | 0.9356 |  | Estimated |
| **140** | pSrc-RhoGEF -> pSrc + pRhoGEF |  |  | 10 | Estimated |
| **141** | pRhoGEF -> RhoGEF |  |  | 0.1298 | Estimated |
| **142** | pSrc + RhoGAP = pSrc-RhoGAP | 40 | 0.9356 |  | Estimated |
| **143** | pSrc-RhoGAP -> pSrc + pRhoGAP |  |  | 10 | Estimated |
| **144** | pRhoGAP + RhoGTP = pRhoGAP-RhoGTP | 2.845 | 0.96 |  | 9535855 |
| **145** | pRhoGAP-RhoGTP -> pRhoGAP + RhoGDP |  |  | 1.205 | 9535855 |
| **146** | RhoGTP + ROCK = RhoGTP-ROCK | 1.754 | 0.5 |  | Estimated |
| **147** | RhoGTP-ROCK -> RhoGTP + pROCK |  |  | 7.624 | Estimated |
| **148** | EGF-pEGFR-2 + Src = EGF-pEGFR-2-Src | 40 | 0.9356 |  | 14751248 |
| **149** | EGF-pEGFR-2-Src -> EGF-pEGFR-2-pSrc |  |  | 40 | 14751248 |
| **150** | EGF-pEGFR-2-pSrc = EGF-pEGRF-2 + pSrc | 0.0003302 | 1 |  | 14751248 |
| **151** | pSrc + TP7 = pSrc-TP7 | 1 | 1 |  | 16687399 |
| **152** | pSrc-TP7 -> Src-TP7 |  |  | 0.01 | 16687399 |
| **153** | Src-TP7 = Src + TP7 | 1.2987 | 0.1 |  | 16687399 |
|  | ***Endocytosis, degradation, recycling of EGFR*** |  |  |  |  |
| **154** | EGF-pEGFR-2-pShc-Grb2-SOS + Cbl-CIN85 = EGF-pEGFR-2-pShc-Grb2-SOS-Cbl-CIN85 | 0.5 | 0.005 |  | 14751248 |
| **155** | EGF-pEGFR-2-Grb2-SOS + Cbl-CIN85 = EGF-pEGFR-2-Grb2-SOS-Cbl-CIN85 | 0.5 | 0.005 |  | 14751248 |
| **156** | EGF-pEGFR-2-pShc-Grb2-SOS-Cbl-CIN85 + EPn = EGF-pEGFR-2-pShc-Grb2-SOS-Cbl-CIN85-EPn | 5 | 0.01 |  | 14751248 |
| **157** | EGF-pEGFR-2-Grb2-SOS-Cbl-CIN85 + EPn = EGF-pEGFR-2-Grb2-SOS-Cbl-CIN85-EPn | 5 | 0.01 |  | 14751248 |
| **158** | EGF-pEGFR-2-pShc-Grb2-SOS-Cbl-CIN85-EPn -> EGF-pEGFR-2-pShc-Grb2-SOS_e + Cbl-CIN85 + EPn |  |  | 0.1 | 14751248 |
| **159** | EGF-pEGFR-2-Grb2-SOS-Cbl-CIN85-EPn -> EGF-pEGFR-2-Grb2-SOS_e + Cbl-CIN85 + EPn |  |  | 0.1 | 14751248 |
|  | ***Signaling from internalized EGFR to Raf on late endosomes*** |  |  |  |  |
| **160** | EGF-pEGFR-2_e -> EGF-pEGFR-2-degrade |  |  | 0.1 | 14751248 |
| **161** | EGF-pEGFR-2_e + SHP_e = EGF-pEGFR-2-SHP_e | 3.14 | 0.2 |  | 14751248 |
| **162** | EGF-pEGFR-2-SHP_e -> EGF-EGFR-2_e + SHP_e |  |  | 0.2661 | 14751248 |
| **163** | EGF-pEGFR-2_e + Shc_e = EGF-pEGFR-2-Shc_e | 90 | 0.6 |  | 10514507; 14751248 |
| **164** | EGF-pEGFR-2-Shc_e -> EGF-pEGFR-2-pShc_e |  |  | 0.5838 | 14751248 |
| **165** | EGF-pEGFR-2-pShc_e = EGF-pEGFR-2_e + pShc_e | 0.3 | 4.481 |  | 14751248 |
| **166** | pShc_e + SHP_e = pShc-SHP_e | 3.114 | 0.2 |  | 14751248 |
| **167** | pShc-SHP_e -> Shc_e + SHP_e |  |  | 0.2661 | 14751248 |
| **168** | EGF-pEGFR-2-pShc_e + Grb2_e = EGF-pEGFR-2-pShc-Grb2_e | 3 | 0.1 |  | 10514507 |
| **169** | EGF-pEGFR-2-pShc-Grb2_e + SOS_e = EGF-pEGFR-2-pShc-Grb2-SOS_e | 10 | 0.0214 |  | 10514507 |
| **170** | Grb2_e + SOS_e = Grb2-SOS_e | 0.1 | 0.0015 |  | 10514507 |
| **171** | EGF-pEGFR-2-pShc_e + Grb2-SOS_e = EGF-pEGFR-2-pShc-Grb2-SOS_e | 10 | 0.045 |  | 15793571; 14571248 |
| **172** | EGF-pEGFR-2-pShc-Grb2-SOS_e + RasGDP_e = EGF-pEGFR-2-pShc-Grb2-SOS-RasGDP_e | 202.9 | 0.18 |  | 14751248 |
| **173** | EGF-pEGFR-2-pShc-Grb2-SOS-RasGDP_e -> EGF-pEGFR-2-pShc-Grb2-SOS_e + RasGTP_e |  |  | 40 | 14751248 |
| **174** | EGF-pEGFR-2_e + Grb2_e = EGF-pEGFR-2-Grb2_e | 3 | 0.05 |  | 10514507 |
| **175** | EGF-pEGFR-2-Grb2_e + SOS_e = EGF-pEGFR-2-Grb2-SOS_e | 10 | 0.06 |  | 10514507 |
| **176** | EGF-pEGFR-2_e + Grb2-SOS_e = EGF-pEGFR-2-Grb2-SOS_e | 2.734 | 0.025 |  | 14751248 |
| **177** | EGF-pEGFR-2-Grb2-SOS_e + RasGDP_e = EGF-pEGFR-2-Grb2-SOS-RasGDP_e | 202.9 | 0.18 |  | 14751248 |
| **178** | EGF-pEGFR-2-Grb2-SOS-RasGDP_e -> EGF-pEGFR-2-Grb2-SOS_e + RasGTP_e |  |  | 0.1434 | 14751248 |
| **179** | Raf_e + RasGTP_e = Raf-RasGTP_e | 1.754 | 0.05 |  | 14751248 |
| **180** | Raf-RasGTP_e -> pRaf_e + RasGTP_e |  |  | 0.7624 | 14751248 |
| **181** | pRaf_e + Pase_e = pRaf-Pase_e | 71.7 | 0.2 |  | 11923843 |
| **182** | pRaf-Pase_e -> Raf_e + Pase_e |  |  | 1 | 11923843 |
| **183** | ppMEK1_e + PP2A_e = ppMEK1-PP2A_e | 14.3 | 0.8 |  | 11923843 |
| **184** | ppMEK1-PP2A_e -> pMEK1_e + PP2A_e |  |  | 0.058 | 11923843 |
| **185** | pMEK1_e + PP2A_e = pMEK1-PP2A_e | 0.25 | 0.5 |  | 11923843 |
| **186** | pMEK1-PP2A_e -> MEK1_e + PP2A_e |  |  | 0.058 | 11923843 |
| **187** | ppERK1_e + MKP3_e = ppERK1-MKP3_e | 7 | 0.6 |  | 11923843 |
| **188** | ppERK1-MKP3_e -> pERK1_e + MKP3_e |  |  | 0.27 | 11923843 |
| **189** | pERK1_e + MKP3_e = pERK1-MKP3_e | 5 | 0.5 |  | 11923843 |
| **190** | pERK1-MKP3_e -> ERK1_e + MKP3_e |  |  | 0.3 | 11923843 |
| **191** | ppERK1_m_e + MKP3_e = ppERK1_m-MKP3_e | 7 | 0.6 |  | 11923843 |
| **192** | ppERK1_m-MKP3_e -> pERK1_e + MKP3_e |  |  | 0.27 | 11923843 |
| **193** | ppMEK2_e + PP2A_e = ppMEK2-PP2A_e | 14.3 | 0.8 |  | 11923843 |
| **194** | ppMEK2-PP2A_e -> pMEK2_e + PP2A_e |  |  | 0.058 | 11923843 |
| **195** | pMEK2_e + PP2A_e = pMEK2-PP2A_e | 0.25 | 0.5 |  | 11923843 |
| **196** | pMEK2-PP2A_e -> MEK2_e + PP2A_e |  |  | 0.058 | 11923843 |
| **197** | ppERK2_e + MKP3_e = ppERK2-MKP3_e | 7 | 0.6 |  | 11923843 |
| **198** | ppERK2-MKP3_e -> pERK2_e + MKP3_e |  |  | 0.27 | 11923843 |
| **199** | pERK2_e + MKP3_e = pERK2-MKP3_e | 5 | 0.5 |  | 11923843 |
| **200** | pERK2-MKP3_e -> ERK2_e + MKP3_e |  |  | 0.3 | 11923843 |
| **201** | RasGTP_e -> RasGDP_e |  |  | 1.667E-05 | 14751248 |
| **202** | RasGTP_e + RasGAP_e = RasGTP-RasGAP_e | 2.854 | 0.96 |  | 14751248 |
| **203** | RasGTP-RasGAP_e -> RasGDP_e + RasGAP_e |  |  | 7.6 | 14751248 |
| **204** | ppERK1_e + EGF-pEGFR-2-pShc-Grb2-SOS_e = ppERK1-EGF-pEGFR-2-pShc-Grb2-SOS_e | 8.898 | 0.1 |  | 14751248 |
| **205** | ppERK2_e + EGF-pEGFR-2-pShc-Grb2-SOS_e = ppERK2-EGF-pEGFR-2-pShc-Grb2-SOS_e | 8.898 | 0.1 |  | 14751248 |
| **206** | ppERK1-EGF-pEGFR-2-pShc-Grb2-SOS_e -> ppERK1_e + EGF-pEGFR-2_e + pShc_e + Grb2_e + pSOS_e |  |  | 0.426 | 14751248 |
| **207** | ppERK2-EGF-pEGFR-2-pShc-Grb2-SOS_e -> ppERK2_e + EGF-pEGFR-2_e + pShc_e + Grb2_e + pSOS_e |  |  | 0.426 | 14751248 |
| **208** | ppERK1_m_e + EGF-pEGFR-2-pShc-Grb2-SOS_e = ppERK1_m-EGF-pEGFR-2-pShc-Grb2-SOS_e | 8.898 | 0.1 |  | 14751248 |
| **209** | ppERK1_m-EGF-pEGFR-2-pShc-Grb2-SOS_e -> ppERK1_m_e + EGF-pEGFR-2_e + pShc_e + Grb2_e + pSOS_e |  |  | 0.426 | 14751248 |
| **210** | ppERK1_e + EGF-pEGFR-2-Grb2-SOS_e = ppERK1-EGF-pEGFR-2-Grb2-SOS_e | 8.898 | 0.1 |  | 14751248 |
| **211** | ppERK2_e + EGF-pEGFR-2-Grb2-SOS_e = ppERK2-EGF-pEGFR-2-Grb2-SOS_e | 8.898 | 0.1 |  | 14751248 |
| **212** | ppERK1-EGF-pEGFR-2-Grb2-SOS_e -> ppERK1_e + EGF-pEGFR-2 + Grb2 + pSOS_e |  |  | 0.426 | 14751248 |
| **213** | ppERK2-EGF-pEGFR-2-Grb2-SOS_e -> ppERK2_e + EGF-pEGFR-2 + Grb2 + pSOS_e |  |  | 0.426 | 14751248 |
| **214** | ppERK1_m_e + EGF-pEGFR-2-Grb2-SOS_e = ppERK1_m-EGF-pEGFR-2-Grb2-SOS_e | 8.898 | 0.1 |  | 14751248 |
| **215** | ppERK1_m-EGF-pEGFR-2-Grb2-SOS_e -> ppERK1_m_e + EGF-pEGFR-2 + Grb2 + pSOS_e |  |  | 0.426 | 14751248 |
| **216** | pSOS_e -> SOS_e |  |  | 0.002 | 15793571 |
| **217** | EGF-pEGFR-2_e + RasGAP_e = EGF-pEGFR-2-RasGAP_e | 0.1 | 0.01 |  | 14751248 |
| **218** | EGF-pEGFR-2-RasGAP_e + RasGTP_e = EGF-pEGFR-2-RasGAP-RasGTP_e | 2.854 | 0.96 |  | 14751248 |
| **219** | EGF-pEGFR-2-RasGAP-RasGTP_e -> EGF-pEGFR-2-RasGAP_e + RasGDP_e |  |  | 7.76 | 14751248 |
| **220** | EGF-pEGFR-2-pShc-Grb2_e + SHP2_e = EGF-pEGFR-2-pShc-Grb2-SHP2_e | 10 | 1 |  | 14751248 |
| **221** | EGF-pEGFR-2-Grb2_e + SHP2_e = EGF-pEGFR-2-Grb2-SHP2_e | 10 | 1 |  | 14751248 |
| **222** | EGF-pEGFR-2-pShc-Grb2-SHP2_e -> EGF-EGFR-2_e + pShc_e + Grb2_e + SHP2_e |  |  | 2.661 | 14751248 |
| **223** | EGF-pEGFR-2-Grb2-SHP2_e -> EGF-EGFR-2_e + Grb2_e + SHP2_e |  |  | 2.661 | 14751248 |
| **224** | EGF-pEGFR-2-RasGAP_e + SHP2_e = EGF-pEGFR-2-RasGAP-SHP2_e | 3.114 | 0.2 |  | 14751248 |
| **225** | EGF-pEGFR-2-RasGAP-SHP2_e -> EGF-EGFR-2_e + RasGAP_e + SHP2_e |  |  | 2.661 | 14751248 |
| **226** | Pro-EGFR -> EGFR |  |  | 0.005 | 14751248 |
| **227** | pROCK + EPn = pROCK-EPn | 1.1 | 0.033 |  | Estimated |
| **228** | pROCK-EPn -> pROCK + pEPn |  |  | 16 | Estimated |
| **229** | pEPn + MPase = pEPn-Mpase | 10 | 0.005 |  | Estimated |
| **230** | pEPn-Mpase -> EPn + Mpase |  |  | 500000 | Estimated |
| **231** | RasGTP + RhoGEF = RasGTP-RhoGEF | 1.754 | 0.05 |  | Estimated |
| **232** | RasGTP-RhoGEF -> RasGTP + pRhoGEF |  |  | 0.07624 | Estimated |
| **233** | ppERK1 + pROCK = ppERK1-pROCK | 8.898 | 1 |  | Estimated |
| **234** | ppERK1-pROCK -> ppERK1 + ROCK |  |  | 0.426 | Estimated |
| **235** | ppERK2 + pROCK = ppERK2-pROCK | 8.898 | 1 |  | Estimated |
| **236** | ppERK2-pROCK -> ppERK2 + ROCK |  |  | 0.426 | Estimated |
| **237** | ppERK1_k + pROCK = ppERK1_k-pROCK | 8.898 | 1 |  | Estimated |
| **238** | ppERK1_k-pROCK -> ppERK1_k + ROCK |  |  | 0.426 | Estimated |
| **239** | ppERK2_k + pROCK = ppERK2_k-pROCK | 8.898 | 1 |  | Estimated |
| **240** | ppERK2_k-pROCK -> ppERK2_k + ROCK |  |  | 0.426 | Estimated |
| **241** | ppERK1_m_e + pROCK = ppERK1_m_e-pROCK | 8.898 | 1 |  | Estimated |
| **242** | ppERK1_m_e-pROCK -> ppERK1_m_e + ROCK |  |  | 0.426 | Estimated |
|  | ***KSR-mediated Raf-MEK-ERK signaling on endosomes*** |  |  |  |  |
| **243** | KSR01a0 + Raf = KSR11a0 | 10 | 0.05 |  | 10823939 |
| **244** | KSR01b0 + Raf = KSR11b0 | 10 | 0.05 |  | 10823939 |
| **245** | KSR01a1a + Raf = KSR11a1a | 10 | 0.05 |  | 10823939 |
| **246** | KSR01a1b + Raf = KSR11a1b | 10 | 0.05 |  | 10823939 |
| **247** | KSR01b1a + Raf = KSR11b1a | 10 | 0.05 |  | 10823939 |
| **248** | KSR01b1b + Raf = KSR11b1b | 10 | 0.05 |  | 10823939 |
| **249** | KSR01a2a + Raf = KSR11a2a | 10 | 0.05 |  | 10823939 |
| **250** | KSR01a2b + Raf = KSR11a2b | 10 | 0.05 |  | 10823939 |
| **251** | KSR01b2a + Raf = KSR11b2a | 10 | 0.05 |  | 10823939 |
| **252** | KSR01b2b + Raf = KSR11b2b | 10 | 0.05 |  | 10823939 |
| **253** | KSR02a0 + Raf = KSR12a0 | 10 | 0.05 |  | 10823939 |
| **254** | KSR02b0 + Raf = KSR12b0 | 10 | 0.05 |  | 10823939 |
| **255** | KSR02a1a + Raf = KSR12a1a | 10 | 0.05 |  | 10823939 |
| **256** | KSR02a1b + Raf = KSR12a1b | 10 | 0.05 |  | 10823939 |
| **257** | KSR02b1a + Raf = KSR12b1a | 10 | 0.05 |  | 10823939 |
| **258** | KSR02b1b + Raf = KSR12b1b | 10 | 0.05 |  | 10823939 |
| **259** | KSR02a2a + Raf = KSR12a2a | 10 | 0.05 |  | 10823939 |
| **260** | KSR02a2b + Raf = KSR12a2b | 10 | 0.05 |  | 10823939 |
| **261** | KSR02b2a + Raf = KSR12b2a | 10 | 0.05 |  | 10823939 |
| **262** | KSR02b2b + Raf = KSR12b2b | 10 | 0.05 |  | 10823939 |
| **263** | KSR000 + Raf = KSR100 | 10 | 0.05 |  | 10823939 |
| **264** | KSR001a + Raf = KSR101a | 10 | 0.05 |  | 10823939 |
| **265** | KSR001b + Raf = KSR101b | 10 | 0.05 |  | 10823939 |
| **266** | KSR002a + Raf = KSR102a | 10 | 0.05 |  | 10823939 |
| **267** | KSR002b + Raf = KSR102b | 10 | 0.05 |  | 10823939 |
| **268** | KSR000 + MEK1 = KSR01a0 | 10 | 0.05 |  | 10823939 |
| **269** | KSR000 + MEK2 = KSR01b0 | 10 | 0.05 |  | 10823939 |
| **270** | KSR001a + MEK1 = KSR01a1a | 10 | 0.05 |  | 10823939 |
| **271** | KSR001b + MEK1 = KSR01a1b | 10 | 0.05 |  | 10823939 |
| **272** | KSR001a + MEK2 = KSR01b1a | 10 | 0.05 |  | 10823939 |
| **273** | KSR001b + MEK2 = KSR01b1b | 10 | 0.05 |  | 10823939 |
| **274** | KSR002a + MEK1 = KSR01a2a | 10 | 0.05 |  | 10823939 |
| **275** | KSR002b + MEK1 = KSR01a2b | 10 | 0.05 |  | 10823939 |
| **276** | KSR002a + MEK2 = KSR01b2a | 10 | 0.05 |  | 10823939 |
| **277** | KSR002b + MEK2 = KSR01b2b | 10 | 0.05 |  | 10823939 |
| **278** | KSR100 + MEK1 = KSR11a0 | 10 | 0.05 |  | 10823939 |
| **279** | KSR100 + MEK2 = KSR11b0 | 10 | 0.05 |  | 10823939 |
| **280** | KSR101a + MEK1 = KSR11a1a | 10 | 0.05 |  | 10823939 |
| **281** | KSR101b + MEK1 = KSR11a1b | 10 | 0.05 |  | 10823939 |
| **282** | KSR101a + MEK2 = KSR11b1a | 10 | 0.05 |  | 10823939 |
| **283** | KSR101b + MEK2 = KSR11b1b | 10 | 0.05 |  | 10823939 |
| **284** | KSR102a + MEK1 = KSR11a2a | 10 | 0.05 |  | 10823939 |
| **285** | KSR102b + MEK1 = KSR11a2b | 10 | 0.05 |  | 10823939 |
| **286** | KSR102a + MEK2 = KSR11b2a | 10 | 0.05 |  | 10823939 |
| **287** | KSR102b + MEK2 = KSR11b2b | 10 | 0.05 |  | 10823939 |
| **288** | KSR200 + MEK1 = KSR21a0 | 10 | 0.05 |  | 10823939 |
| **289** | KSR200 + MEK2 = KSR21b0 | 10 | 0.05 |  | 10823939 |
| **290** | KSR201a + MEK1 = KSR21a1a | 10 | 0.05 |  | 10823939 |
| **291** | KSR201b + MEK1 = KSR21a1b | 10 | 0.05 |  | 10823939 |
| **292** | KSR201a + MEK2 = KSR21b1a | 10 | 0.05 |  | 10823939 |
| **293** | KSR201b + MEK2 = KSR21b1b | 10 | 0.05 |  | 10823939 |
| **294** | KSR202a + MEK1 = KSR21a2a | 10 | 0.05 |  | 10823939 |
| **295** | KSR202b + MEK1 = KSR21a2b | 10 | 0.05 |  | 10823939 |
| **296** | KSR202a + MEK2 = KSR21b2a | 10 | 0.05 |  | 10823939 |
| **297** | KSR202b + MEK2 = KSR21b2b | 10 | 0.05 |  | 10823939 |
| **298** | KSR01a0 + ERK1 = KSR01a1a | 10 | 0.05 |  | 10823939 |
| **299** | KSR01b0 + ERK1 = KSR01b1a | 10 | 0.05 |  | 10823939 |
| **300** | KSR01a0 + ERK2 = KSR01a1b | 10 | 0.05 |  | 10823939 |
| **301** | KSR01b0 + ERK2 = KSR01b1b | 10 | 0.05 |  | 10823939 |
| **302** | KSR11a0 + ERK1 = KSR11a1a | 10 | 0.05 |  | 10823939 |
| **303** | KSR11b0 + ERK1 = KSR11b1a | 10 | 0.05 |  | 10823939 |
| **304** | KSR11a0 + ERK2 = KSR11a1b | 10 | 0.05 |  | 10823939 |
| **305** | KSR11b0 + ERK2 = KSR11b1b | 10 | 0.05 |  | 10823939 |
| **306** | KSR21a0 + ERK1 = KSR21a1a | 10 | 0.05 |  | 10823939 |
| **307** | KSR21b0 + ERK1 = KSR21b1a | 10 | 0.05 |  | 10823939 |
| **308** | KSR21a0 + ERK2 = KSR21a1b | 10 | 0.05 |  | 10823939 |
| **309** | KSR21b0 + ERK2 = KSR21b1b | 10 | 0.05 |  | 10823939 |
| **310** | KSR02a0 + ERK1 = KSR02a1a | 10 | 0.05 |  | 10823939 |
| **311** | KSR02b0 + ERK1 = KSR02b1a | 10 | 0.05 |  | 10823939 |
| **312** | KSR02a0 + ERK2 = KSR02a1b | 10 | 0.05 |  | 10823939 |
| **313** | KSR02b0 + ERK2 = KSR02b1b | 10 | 0.05 |  | 10823939 |
| **314** | KSR12a0 + ERK1 = KSR12a1a | 10 | 0.05 |  | 10823939 |
| **315** | KSR12b0 + ERK1 = KSR12b1a | 10 | 0.05 |  | 10823939 |
| **316** | KSR12a0 + ERK2 = KSR12a1b | 10 | 0.05 |  | 10823939 |
| **317** | KSR12b0 + ERK2 = KSR12b1b | 10 | 0.05 |  | 10823939 |
| **318** | KSR22a0 + ERK1 = KSR22a1a | 10 | 0.05 |  | 10823939 |
| **319** | KSR22b0 + ERK1 = KSR22b1a | 10 | 0.05 |  | 10823939 |
| **320** | KSR22a0 + ERK2 = KSR22a1b | 10 | 0.05 |  | 10823939 |
| **321** | KSR22b0 + ERK2 = KSR22b1b | 10 | 0.05 |  | 10823939 |
| **322** | KSR000 + ERK1 = KSR001a | 10 | 0.05 |  | 10823939 |
| **323** | KSR000 + ERK2 = KSR001b | 10 | 0.05 |  | 10823939 |
| **324** | KSR100 + ERK1 = KSR101a | 10 | 0.05 |  | 10823939 |
| **325** | KSR100 + ERK2 = KSR101b | 10 | 0.05 |  | 10823939 |
| **326** | KSR200 + ERK1 = KSR201a | 10 | 0.05 |  | 10823939 |
| **327** | KSR200 + ERK2 = KSR201b | 10 | 0.05 |  | 10823939 |
| **328** | KSR21a0 -> KSR01a0 + pRaf |  |  | 0.05 | 10823939 |
| **329** | KSR21b0 -> KSR01b0 + pRaf |  |  | 0.05 | 10823939 |
| **330** | KSR21a1a -> KSR01a1a + pRaf |  |  | 0.05 | 10823939 |
| **331** | KSR21a1b -> KSR01a1b + pRaf |  |  | 0.05 | 10823939 |
| **332** | KSR21b1a -> KSR01b1a + pRaf |  |  | 0.05 | 10823939 |
| **333** | KSR21b1b -> KSR01b1b + pRaf |  |  | 0.05 | 10823939 |
| **334** | KSR21a2a -> KSR01a2a + pRaf |  |  | 0.05 | 10823939 |
| **335** | KSR21a2b -> KSR01a2b + pRaf |  |  | 0.05 | 10823939 |
| **336** | KSR21b2a -> KSR01b2a + pRaf |  |  | 0.05 | 10823939 |
| **337** | KSR21b2b -> KSR01b2b + pRaf |  |  | 0.05 | 10823939 |
| **338** | KSR22a0 -> KSR02a0 + pRaf |  |  | 0.05 | 10823939 |
| **339** | KSR22b0 -> KSR02b0 + pRaf |  |  | 0.05 | 10823939 |
| **340** | KSR22a1a -> KSR02a1a + pRaf |  |  | 0.05 | 10823939 |
| **341** | KSR22a1b -> KSR02a1b + pRaf |  |  | 0.05 | 10823939 |
| **342** | KSR22b1a -> KSR02b1a + pRaf |  |  | 0.05 | 10823939 |
| **343** | KSR22b1b -> KSR02b1b + pRaf |  |  | 0.05 | 10823939 |
| **344** | KSR22a2a -> KSR02a2a + pRaf |  |  | 0.05 | 10823939 |
| **345** | KSR22a2b -> KSR02a2b + pRaf |  |  | 0.05 | 10823939 |
| **346** | KSR22b2a -> KSR02b2a + pRaf |  |  | 0.05 | 10823939 |
| **347** | KSR22b2b -> KSR02b2b + pRaf |  |  | 0.05 | 10823939 |
| **348** | KSR200 -> KSR000 + pRaf |  |  | 0.05 | 10823939 |
| **349** | KSR201a -> KSR001a + pRaf |  |  | 0.05 | 10823939 |
| **350** | KSR201b -> KSR001b + pRaf |  |  | 0.05 | 10823939 |
| **351** | KSR202a -> KSR002a + pRaf |  |  | 0.05 | 10823939 |
| **352** | KSR202b -> KSR002b + pRaf |  |  | 0.05 | 10823939 |
| **353** | KSR02a0 -> KSR000 + ppMEK1 |  |  | 0.05 | 10823939 |
| **354** | KSR02b0 -> KSR000 + ppMEK2 |  |  | 0.05 | 10823939 |
| **355** | KSR12a0 -> KSR100 + ppMEK1 |  |  | 0.05 | 10823939 |
| **356** | KSR12b0 -> KSR100 + ppMEK2 |  |  | 0.05 | 10823939 |
| **357** | KSR22a0 -> KSR200 + ppMEK1 |  |  | 0.05 | 10823939 |
| **358** | KSR22b0 -> KSR200 + ppMEK2 |  |  | 0.05 | 10823939 |
| **359** | KSR02a1a -> KSR001a + ppMEK1 |  |  | 0.05 | 10823939 |
| **360** | KSR02a1b -> KSR001b + ppMEK1 |  |  | 0.05 | 10823939 |
| **361** | KSR02b1a -> KSR001a + ppMEK2 |  |  | 0.05 | 10823939 |
| **362** | KSR02b1b -> KSR001b + ppMEK2 |  |  | 0.05 | 10823939 |
| **363** | KSR02a2a -> KSR002a + ppMEK1 |  |  | 0.05 | 10823939 |
| **364** | KSR02a2b -> KSR002b + ppMEK1 |  |  | 0.05 | 10823939 |
| **365** | KSR02b2a -> KSR002a + ppMEK2 |  |  | 0.05 | 10823939 |
| **366** | KSR02b2b -> KSR002b + ppMEK2 |  |  | 0.05 | 10823939 |
| **367** | KSR12a1a -> KSR101a + ppMEK1 |  |  | 0.05 | 10823939 |
| **368** | KSR12a1b -> KSR101b + ppMEK1 |  |  | 0.05 | 10823939 |
| **369** | KSR12b1a -> KSR101a + ppMEK2 |  |  | 0.05 | 10823939 |
| **370** | KSR12b1b -> KSR101b + ppMEK2 |  |  | 0.05 | 10823939 |
| **371** | KSR12a2a -> KSR102a + ppMEK1 |  |  | 0.05 | 10823939 |
| **372** | KSR12a2b -> KSR102b + ppMEK1 |  |  | 0.05 | 10823939 |
| **373** | KSR12b2a -> KSR102a + ppMEK2 |  |  | 0.05 | 10823939 |
| **374** | KSR12b2b -> KSR102b + ppMEK2 |  |  | 0.05 | 10823939 |
| **375** | KSR22a1a -> KSR201a + ppMEK1 |  |  | 0.05 | 10823939 |
| **376** | KSR22a1b -> KSR201b + ppMEK1 |  |  | 0.05 | 10823939 |
| **377** | KSR22b1a -> KSR201a + ppMEK2 |  |  | 0.05 | 10823939 |
| **378** | KSR22b1b -> KSR201b + ppMEK2 |  |  | 0.05 | 10823939 |
| **379** | KSR22a2a -> KSR202a + ppMEK1 |  |  | 0.05 | 10823939 |
| **380** | KSR22a2b -> KSR202b + ppMEK1 |  |  | 0.05 | 10823939 |
| **381** | KSR22b2a -> KSR202a + ppMEK2 |  |  | 0.05 | 10823939 |
| **382** | KSR22b2b -> KSR202b + ppMEK2 |  |  | 0.05 | 10823939 |
| **383** | KSR01a2a -> KSR01a0 + ppERK1_k |  |  | 0.5 | 10823939 |
| **384** | KSR01b2a -> KSR01b0 + ppERK1_k |  |  | 0.5 | 10823939 |
| **385** | KSR01a2b -> KSR01a0 + ppERK2_k |  |  | 0.5 | 10823939 |
| **386** | KSR01b2b -> KSR01b0 + ppERK2_k |  |  | 0.5 | 10823939 |
| **387** | KSR21a2a -> KSR21a0 + ppERK1_k |  |  | 0.5 | 10823939 |
| **388** | KSR21b2a -> KSR21b0 + ppERK1_k |  |  | 0.5 | 10823939 |
| **389** | KSR21a2b -> KSR21a0 + ppERK2_k |  |  | 0.5 | 10823939 |
| **390** | KSR21b2b -> KSR21b0 + ppERK2_k |  |  | 0.5 | 10823939 |
| **391** | KSR11a2a -> KSR11a0 + ppERK1_k |  |  | 0.5 | 10823939 |
| **392** | KSR11b2a -> KSR11b0 + ppERK1_k |  |  | 0.5 | 10823939 |
| **393** | KSR11a2b -> KSR11a0 + ppERK2_k |  |  | 0.5 | 10823939 |
| **394** | KSR11b2b -> KSR11b0 + ppERK2_k |  |  | 0.5 | 10823939 |
| **395** | KSR02a2a -> KSR02a0 + ppERK1_k |  |  | 0.5 | 10823939 |
| **396** | KSR02b2a -> KSR02b0 + ppERK1_k |  |  | 0.5 | 10823939 |
| **397** | KSR02a2b -> KSR02a0 + ppERK2_k |  |  | 0.5 | 10823939 |
| **398** | KSR02b2b -> KSR02b0 + ppERK2_k |  |  | 0.5 | 10823939 |
| **399** | KSR12a2a -> KSR12a0 + ppERK1_k |  |  | 0.5 | 10823939 |
| **400** | KSR12b2a -> KSR12b0 + ppERK1_k |  |  | 0.5 | 10823939 |
| **401** | KSR12a2b -> KSR12a0 + ppERK2_k |  |  | 0.5 | 10823939 |
| **402** | KSR12b2b -> KSR12b0 + ppERK2_k |  |  | 0.5 | 10823939 |
| **403** | KSR22a2a -> KSR22a0 + ppERK1_k |  |  | 0.5 | 10823939 |
| **404** | KSR22b2a -> KSR22b0 + ppERK1_k |  |  | 0.5 | 10823939 |
| **405** | KSR22a2b -> KSR22a0 + ppERK2_k |  |  | 0.5 | 10823939 |
| **406** | KSR22b2b -> KSR22b0 + ppERK2_k |  |  | 0.5 | 10823939 |
| **407** | KSR002a -> KSR000 + ppERK1_k |  |  | 0.5 | 10823939 |
| **408** | KSR002b -> KSR000 + ppERK2_k |  |  | 0.5 | 10823939 |
| **409** | KSR102a -> KSR100 + ppERK1_k |  |  | 0.5 | 10823939 |
| **410** | KSR102b -> KSR100 + ppERK2_k |  |  | 0.5 | 10823939 |
| **411** | KSR202a -> KSR200 + ppERK1_k |  |  | 0.5 | 10823939 |
| **412** | KSR202b -> KSR200 + ppERK2_k |  |  | 0.5 | 10823939 |
| **413** | KSR11a0 + RasGTP = RasGTP-KSR11a0 | 1.754 | 0.05 |  | 14751248 |
| **414** | KSR11b0 + RasGTP = RasGTP-KSR11b0 | 1.754 | 0.05 |  | 14751248 |
| **415** | RasGTP-KSR11a0 -> KSR21a0 + RasGTP |  |  | 0.7624 | 14751248 |
| **416** | RasGTP-KSR11b0 -> KSR21b0 + RasGTP |  |  | 0.7624 | 14751248 |
| **417** | KSR11a1a + RasGTP = RasGTP-KSR11a1a | 1.754 | 0.05 |  | 14751248 |
| **418** | KSR11a1b + RasGTP = RasGTP-KSR11a1b | 1.754 | 0.05 |  | 14751248 |
| **419** | KSR11b1a + RasGTP = RasGTP-KSR11b1a | 1.754 | 0.05 |  | 14751248 |
| **420** | KSR11b1b + RasGTP = RasGTP-KSR11b1b | 1.754 | 0.05 |  | 14751248 |
| **421** | RasGTP-KSR11a1a -> KSR21a1a + RasGTP |  |  | 0.7624 | 14751248 |
| **422** | RasGTP-KSR11a1b -> KSR21a1b + RasGTP |  |  | 0.7624 | 14751248 |
| **423** | RasGTP-KSR11b1a -> KSR21b1a + RasGTP |  |  | 0.7624 | 14751248 |
| **424** | RasGTP-KSR11b1b -> KSR21b1b + RasGTP |  |  | 0.7624 | 14751248 |
| **425** | KSR11a2a + RasGTP = RasGTP-KSR11a2a | 1.754 | 0.05 |  | 14751248 |
| **426** | KSR11a2b + RasGTP = RasGTP-KSR11a2b | 1.754 | 0.05 |  | 14751248 |
| **427** | KSR11b2a + RasGTP = RasGTP-KSR11b2a | 1.754 | 0.05 |  | 14751248 |
| **428** | KSR11b2b + RasGTP = RasGTP-KSR11b2b | 1.754 | 0.05 |  | 14751248 |
| **429** | RasGTP-KSR11a2a -> KSR21a2a + RasGTP |  |  | 0.7624 | 14751248 |
| **430** | RasGTP-KSR11a2b -> KSR21a2b + RasGTP |  |  | 0.7624 | 14751248 |
| **431** | RasGTP-KSR11b2a -> KSR21b2a + RasGTP |  |  | 0.7624 | 14751248 |
| **432** | RasGTP-KSR11b2b -> KSR21b2b + RasGTP |  |  | 0.7624 | 14751248 |
| **433** | KSR12a0 + RasGTP = RasGTP-KSR12a0 | 1.754 | 0.05 |  | 14751248 |
| **434** | KSR12b0 + RasGTP = RasGTP-KSR12b0 | 1.754 | 0.05 |  | 14751248 |
| **435** | RasGTP-KSR12a0 -> KSR22a0 + RasGTP |  |  | 0.7624 | 14751248 |
| **436** | RasGTP-KSR12b0 -> KSR22b0 + RasGTP |  |  | 0.7624 | 14751248 |
| **437** | KSR12a1a + RasGTP = RasGTP-KSR12a1a | 1.754 | 0.05 |  | 14751248 |
| **438** | KSR12a1b + RasGTP = RasGTP-KSR12a1b | 1.754 | 0.05 |  | 14751248 |
| **439** | KSR12b1a + RasGTP = RasGTP-KSR12b1a | 1.754 | 0.05 |  | 14751248 |
| **440** | KSR12b1b + RasGTP = RasGTP-KSR12b1b | 1.754 | 0.05 |  | 14751248 |
| **441** | RasGTP-KSR12a1a -> KSR22a1a + RasGTP |  |  | 0.7624 | 14751248 |
| **442** | RasGTP-KSR12a1b -> KSR22a1b + RasGTP |  |  | 0.7624 | 14751248 |
| **443** | RasGTP-KSR12b1a -> KSR22b1a + RasGTP |  |  | 0.7624 | 14751248 |
| **444** | RasGTP-KSR12b1b -> KSR22b1b + RasGTP |  |  | 0.7624 | 14751248 |
| **445** | KSR12a2a + RasGTP = RasGTP-KSR12a2a | 1.754 | 0.05 |  | 14751248 |
| **446** | KSR12a2b + RasGTP = RasGTP-KSR12a2b | 1.754 | 0.05 |  | 14751248 |
| **447** | KSR12b2a + RasGTP = RasGTP-KSR12b2a | 1.754 | 0.05 |  | 14751248 |
| **448** | KSR12b2b + RasGTP = RasGTP-KSR12b2b | 1.754 | 0.05 |  | 14751248 |
| **449** | RasGTP-KSR12a2a -> KSR22a2a + RasGTP |  |  | 0.7624 | 14751248 |
| **450** | RasGTP-KSR12a2b -> KSR22a2b + RasGTP |  |  | 0.7624 | 14751248 |
| **451** | RasGTP-KSR12b2a -> KSR22b2a + RasGTP |  |  | 0.7624 | 14751248 |
| **452** | RasGTP-KSR12b2b -> KSR22b2b + RasGTP |  |  | 0.7624 | 14751248 |
| **453** | KSR100 + RasGTP = RasGTP-KSR100 | 1.754 | 0.05 |  | 14751248 |
| **454** | RasGTP-KSR100 -> KSR200 + RasGTP |  |  | 0.7624 | 14751248 |
| **455** | KSR101a + RasGTP = RasGTP-KSR101a | 1.754 | 0.05 |  | 14751248 |
| **456** | KSR101b + RasGTP = RasGTP-KSR101b | 1.754 | 0.05 |  | 14751248 |
| **457** | RasGTP-KSR101a -> KSR201a + RasGTP |  |  | 0.7624 | 14751248 |
| **458** | RasGTP-KSR101b -> KSR201b + RasGTP |  |  | 0.7624 | 14751248 |
| **459** | KSR102a + RasGTP = RasGTP-KSR102a | 1.754 | 0.05 |  | 14751248 |
| **460** | KSR102b + RasGTP = RasGTP-KSR102b | 1.754 | 0.05 |  | 14751248 |
| **461** | RasGTP-KSR102a -> KSR202a + RasGTP |  |  | 0.7624 | 14751248 |
| **462** | RasGTP-KSR102b -> KSR202b + RasGTP |  |  | 0.7624 | 14751248 |
| **463** | KSR21a0 -> KSR22a0 |  |  | 2.9 | 11923843 |
| **464** | KSR21b0 -> KSR22b0 |  |  | 2.9 | 11923843 |
| **465** | KSR21a1a -> KSR22a1a |  |  | 2.9 | 11923843 |
| **466** | KSR21a1b -> KSR22a1b |  |  | 2.9 | 11923843 |
| **467** | KSR21b1a -> KSR22b1a |  |  | 2.9 | 11923843 |
| **468** | KSR21b1b -> KSR22b1b |  |  | 2.9 | 11923843 |
| **469** | KSR21a2a -> KSR22a2a |  |  | 2.9 | 11923843 |
| **470** | KSR21a2b -> KSR22a2b |  |  | 2.9 | 11923843 |
| **471** | KSR21b2a -> KSR22b2a |  |  | 2.9 | 11923843 |
| **472** | KSR21b2b -> KSR22b2b |  |  | 2.9 | 11923843 |
| **473** | KSR02a1a -> KSR02a2a |  |  | 5.7 | 11923843 |
| **474** | KSR02a1b -> KSR02a2b |  |  | 5.7 | 11923843 |
| **475** | KSR02b1a -> KSR02b2a |  |  | 5.7 | 11923843 |
| **476** | KSR02b1b -> KSR02b2b |  |  | 5.7 | 11923843 |
| **477** | KSR12a1a -> KSR12a2a |  |  | 5.7 | 11923843 |
| **478** | KSR12a1b -> KSR12a2b |  |  | 5.7 | 11923843 |
| **479** | KSR12b1a -> KSR12b2a |  |  | 5.7 | 11923843 |
| **480** | KSR12b1b -> KSR12b2b |  |  | 5.7 | 11923843 |
| **481** | KSR22a1a -> KSR22a2a |  |  | 5.7 | 11923843 |
| **482** | KSR22a1b -> KSR22a2b |  |  | 5.7 | 11923843 |
| **483** | KSR22b1a -> KSR22b2a |  |  | 5.7 | 11923843 |
| **484** | KSR22b1b -> KSR22b2b |  |  | 5.7 | 11923843 |
|  | ***Regulation of KSR*** |  |  |  |  |
| **485** | RasGTP + KSR01a0-PP2A-(14-3-3)-IMP = KSR01a0 + PP2A + (14-3-3) + IMP-degrade + RasGTP | 5 | 0.05 |  | 10823939 |
| **486** | RasGTP + KSR01b0-PP2A-(14-3-3)-IMP = KSR01b0 + PP2A + (14-3-3) + IMP-degrade + RasGTP | 5 | 0.05 |  | 10823939 |
|  | ***MP1-mediated Raf-MEK-ERK signaling*** |  |  |  |  |
| **487** | MP1_00 + MEK1_e = MP1_10 | 10 | 0.05 |  | 10823939 |
| **488** | MP1_01 + MEK1_e = MP1_11 | 10 | 0.05 |  | 10823939 |
| **489** | MP1_02 + MEK1_e = MP1_12 | 10 | 0.05 |  | 10823939 |
| **490** | MP1_00 + ERK1_e = MP1_01 | 10 | 0.05 |  | 10823939 |
| **491** | MP1_10 + ERK1_e = MP1_11 | 10 | 0.05 |  | 10823939 |
| **492** | MP1_20 + ERK1_e = MP1_21 | 10 | 0.05 |  | 10823939 |
| **493** | MP1_20 -> MP1_00 + ppMEK1_e |  |  | 0.0005 | Estimated |
| **494** | MP1_21 -> MP1_01 + ppMEK1_e |  |  | 0.0005 | Estimated |
| **495** | MP1_22 -> MP1_02 + ppMEK1_e |  |  | 0.0005 | Estimated |
| **496** | MP1_02 -> MP1_00 + ppERK1_m_e |  |  | 0.5 | 10823939 |
| **497** | MP1_12 -> MP1_10 + ppERK1_m_e |  |  | 0.5 | 10823939 |
| **498** | MP1_22 -> MP1_20 + ppERK1_m_e |  |  | 0.5 | 10823939 |
|  | ***Regulation of MP1*** |  |  |  |  |
| **499** | MP1_02 + p14 = p14-MP1_02 | 0.0468 | 0.000597 |  | 15263099 |
| **500** | MP1_12 + p14 = p14-MP1_12 | 0.0468 | 0.000597 |  | 15263099 |
| **501** | MP1_22 + p14 = p14-MP1_22 | 0.0468 | 0.000597 |  | 15263099 |
| **502** | MP1_00 + p14 = p14-MP1_00 | 0.0468 | 0.000597 |  | 15263099 |
| **503** | MP1_10 + p14 = p14-MP1_10 | 0.0468 | 0.000597 |  | 15263099 |
| **504** | MP1_20 + p14 = p14-MP1_20 | 0.0468 | 0.000597 |  | 15263099 |
| **505** | MP1_01 + p14 = p14-MP1_01 | 0.0468 | 0.000597 |  | 15263099 |
| **506** | MP1_11 + p14 = p14-MP1_11 | 0.0468 | 0.000597 |  | 15263099 |
| **507** | MP1_21 + p14 = p14-MP1_21 | 0.0468 | 0.000597 |  | 15263099 |
| **508** | p14-MP1_00 + p18 = p18-p14-MP1_00 | 0.0468 | 0.000597 |  | 15263099 |
| **509** | p14-MP1_10 + p18 = p18-p14-MP1_10 | 0.0468 | 0.000597 |  | 15263099 |
| **510** | p14-MP1_20 + p18 = p18-p14-MP1_20 | 0.0468 | 0.000597 |  | 15263099 |
| **511** | p14-MP1_01 + p18 = p18-p14-MP1_01 | 0.0468 | 0.000597 |  | 15263099 |
| **512** | p14-MP1_11 + p18 = p18-p14-MP1_11 | 0.0468 | 0.000597 |  | 15263099 |
| **513** | p14-MP1_21 + p18 = p18-p14-MP1_21 | 0.0468 | 0.000597 |  | 15263099 |
| **514** | p14-MP1_02 + p18 = p18-p14-MP1_02 | 0.0468 | 0.000597 |  | 15263099 |
| **515** | p14-MP1_12 + p18 = p18-p14-MP1_12 | 0.0468 | 0.000597 |  | 15263099 |
| **516** | p14-MP1_22 + p18 = p18-p14-MP1_22 | 0.0468 | 0.000597 |  | 15263099 |
| **517** | p18-p14-MP1_02 -> p18-p14-MP1_00 + ppERK1_m_e |  |  | 0.5 | 10823939 |
| **518** | p18-p14-MP1_12 -> p18-p14-MP1_10 + ppERK1_m_e |  |  | 0.5 | 10823939 |
| **519** | p18-p14-MP1_22 -> p18-p14-MP1_20 + ppERK1_m_e |  |  | 0.5 | 10823939 |
| **520** | MP1_10 + pRaf_e = pRaf-MP1_10 | 10 | 0.01833 |  | 11923843 |
| **521** | pRaf-MP1_10 -> MP1_20 + pRaf_e |  |  | 2.9 | 11923843 |
| **522** | MP1_11 + pRaf_e = pRaf-MP1_11 | 10 | 0.01833 |  | 11923843 |
| **523** | pRaf-MP1_11 -> MP1_21 + pRaf_e |  |  | 2.9 | 11923843 |
| **524** | MP1_12 + pRaf_e = pRaf-MP1_12 | 10 | 0.01833 |  | 11923843 |
| **525** | pRaf-MP1_12 -> MP1_22 + pRaf_e |  |  | 2.9 | 11923843 |
| **526** | p18-p14-MP1_00 + MEK1_e = p18-p14-MP1_10 | 50 | 0.05 |  | Estimated |
| **527** | p18-p14-MP1_01 + MEK1_e = p18-p14-MP1_11 | 50 | 0.05 |  | Estimated |
| **528** | p18-p14-MP1_02 + MEK1_e = p18-p14-MP1_12 | 50 | 0.05 |  | Estimated |
| **529** | p18-p14-MP1_00 + ERK1_e = p18-p14-MP1_01 | 50 | 0.05 |  | Estimated |
| **530** | p18-p14-MP1_10 + ERK1_e = p18-p14-MP1_11 | 50 | 0.05 |  | Estimated |
| **531** | p18-p14-MP1_20 + ERK1_e = p18-p14-MP1_21 | 50 | 0.05 |  | Estimated |
| **532** | p18-p14-MP1_20 -> p18-p14-MP1_00 + ppMEK1_e |  |  | 0.0005 | Estimated |
| **533** | p18-p14-MP1_21 -> p18-p14-MP1_01 + ppMEK1_e |  |  | 0.0005 | Estimated |
| **534** | p18-p14-MP1_22 -> p18-p14-MP1_02 + ppMEK1_e |  |  | 0.0005 | Estimated |
| **535** | p18-p14-MP1_10 + pRaf_e = pRaf-p18-p14-MP1_10 | 10 | 0.01833 |  | 11923843 |
| **536** | pRaf-p18-p14-MP1_10 -> p18-p14-MP1_20 + pRaf_e |  |  | 2.9 | 11923843 |
| **537** | p18-p14-MP1_11 + pRaf_e = pRaf-p18-p14-MP1_11 | 10 | 0.01833 |  | 11923843 |
| **538** | pRaf-p18-p14-MP1_11 -> p18-p14-MP1_21 + pRaf_e |  |  | 2.9 | 11923843 |
| **539** | p18-p14-MP1_12 + pRaf_e = pRaf-p18-p14-MP1_12 | 10 | 0.01833 |  | 11923843 |
| **540** | pRaf-p18-p14-MP1_12 -> p18-p14-MP1_22 + pRaf_e |  |  | 2.9 | 11923843 |
| **541** | p18-p14-MP1_21 -> p18-p14-MP1_22 |  |  | 5.7 | 11923843 |

- “_e” denotes species near endosome
- Every member on KSR has multiple states: unbound (0), inactive state (1), active state (2). There are two isoforms for MEK and ERK (denoted as a, b).
- Every member on MP1 has multiple states: unbound (0), inactive state (1), active state (2). Only MEK1 and ERK1 participate in binding onto MP1.
