# Supplementary material for: Simulating EGFR-ERK Signaling Control by Scaffold Proteins KSR and MP1 Reveals Differential Ligand-Sensitivity Co-Regulated by Cbl-CIN85 and Endophilin
Source: PLoS One. 2011 Aug 1;6(8):e22933. doi: 10.1371/journal.pone.0022933 (PMC3148240; doi:10.1371/journal.pone.0022933)
Supplement: Table S2 — List of species and initial concentrations used in the model. (DOC) [file pone.0022933.s009.doc]

**Supplementary Table S2.** List of species and initial concentrations used for the model**.**

| **Initial Concentration** | | | |
| --- | --- | --- | --- |
| **ID** | **Name** | **Concentration (μM)** | **Reference (PMID)** |
| **1** | Akt | 1.00E-01 | 16687399 |
| **2** | Cbl-CIN85 | 8.00E-01 | 15793571 |
| **3** | EGF | 1.66E-02 | 15793571 |
| **4** | EGFR | 3.00E-01 | 15793571 |
| **5** | ERK1 | 2.00E-01 | 15793571 |
| **6** | ERK2 | 2.00E-01 | 15793571 |
| **7** | EPn | 5.00E-01 | 15793571 |
| **8** | Grb2 | 1.00E+00 | 15793571 |
| **9** | MEK1 | 3.40E-01 | 15793571 |
| **10** | MEK2 | 3.40E-01 | 15793571 |
| **11** | MKP3 | 2.00E-03 | 15793571 |
| **12** | MP1_00 | 2.00E-02 | Estimated |
| **13** | Mpase | 1.00E+00 | 15793571 |
| **14** | PDK1 | 1.00E-01 | 16687399 |
| **15** | PI3K | 1.00E-02 | 16687399 |
| **16** | PIP2 | 5.00E-01 | 16687399 |
| **17** | PIP3 | 5.00E-01 | 16687399 |
| **18** | PP2A | 2.00E-02 | 15793571 |
| **19** | PTEN | 1.00E-01 | 16687399 |
| **20** | Pase | 5.00E-01 | 15793571 |
| **21** | Pro-EGFR | 3.00E+03 | 15793571 |
| **22** | ROK | 6.80E-01 | 15793571 |
| **23** | RacGAP | 1.00E-01 | 15793571 |
| **24** | RacGDP | 2.00E-01 | 15793571 |
| **25** | RacGEF | 1.00E-01 | 15793571 |
| **26** | Raf | 5.00E-01 | 15793571 |
| **27** | RasGAP | 1.00E-01 | 15793571 |
| **28** | RasGDP | 1.50E-01 | 15793571 |
| **29** | RhoGAP | 5.00E-01 | 15793571 |
| **30** | RhoGDI | 1.00E-01 | 15793571 |
| **31** | RhoGDP | 1.50E-01 | 15793571 |
| **32** | RhoGEF | 1.00E-01 | 15793571 |
| **33** | SHP | 1.00E-01 | 15793571 |
| **34** | SHP2 | 1.00E-01 | 15793571 |
| **35** | SOS | 3.00E-01 | 15793571 |
| **36** | Shc | 1.00E+00 | 15793571 |
| **37** | Src | 5.18E-01 | 15793571 |
| **38** | TP4 | 2.00E-01 | 16687399 |
| **39** | TP7 | 5.18E-01 | 15793571 |
| **40** | Takt | 1.00E-01 | 16687399 |
| **41** | KSR01a0-PP2A-(14-3-3)-IMP | 1.00E-02 | Estimated |
| **42** | KSR01b0-PP2A-(14-3-3)-IMP | 1.00E-02 | Estimated |
| **43** | p14 | 2.00E-02 | Estimated |
| **44** | p18 | 5.00E-01 | Estimated |
| **45*** | ERK1_e | 2.00E-01 | 15793571 |
| **46*** | ERK2_e | 2.00E-01 | 15793571 |
| **47*** | Grb2_e | 1.00E+00 | 15793571 |
| **48*** | MEK1_e | 3.40E-01 | 15793571 |
| **49*** | MEK2_e | 3.40E-01 | 15793571 |
| **50*** | MKP3_e | 2.00E-03 | 15793571 |
| **51*** | PP2A_e | 2.00E-02 | 15793571 |
| **52*** | Pase_e | 5.00E-01 | 15793571 |
| **53*** | Raf_e | 5.00E-01 | 15793571 |
| **54*** | RasGAP_e | 1.00E-01 | 15793571 |
| **55*** | RasGDP_e | 1.50E-01 | 15793571 |
| **56*** | SHP_e | 1.00E-01 | 15793571 |
| **57*** | SHP2_e | 1.00E-01 | 15793571 |
| **58*** | SOS_e | 3.00E-01 | 15793571 |
| **59*** | Shc_e | 1.00E+00 | 15793571 |

- “_e” denotes species near endosome
- Every member on KSR has multiple states: unbound (0), inactive state (1), active state (2). There are two isoforms for MEK and ERK (denoted as a, b).
- Every member on MP1 has multiple states: unbound (0), inactive state (1), active state (2). Only MEK1 and ERK1 participate in binding onto MP1.
