# Supplementary material for: Simulating EGFR-ERK Signaling Control by Scaffold Proteins KSR and MP1 Reveals Differential Ligand-Sensitivity Co-Regulated by Cbl-CIN85 and Endophilin
Source: PLoS One. 2011 Aug 1;6(8):e22933. doi: 10.1371/journal.pone.0022933 (PMC3148240; doi:10.1371/journal.pone.0022933)
Supplement: Text S1 — A detailed description of the signaling model used in this study. (DOC) [file pone.0022933.s010.doc]

**Supplementary Methods: Model Description**

A detailed description of the signaling model used in this study is given below. The equation numbers in the text refer to the equation numbers given in **Supplementary Table S1**.

***(a) Activation and Inactivation from EGFR to Ras at plasma membrane***

Upon the binding of EGF, the EGF receptor undergoes dimerization and tyrosine autophosphorylation. The phosphorylated tyrosine residues serve as docking sites for various Src homology 2 (SH2) domain- containing adapters such as growth factor receptor binding protein 2 (Grb2) and Src homology 2 and collagen domain protein (Shc) that subsequently recruit rat sarcoma protein (Ras). Ras protein can be activated by both Shc-dependent and Shc-independent mechanisms by activated EGFR dimers.

***(b) Conventional Raf-MEK-ERK signaling in the cytosol (scaffold-independent)***

Activated GTP-bound Ras recruits c-Raf-1, to the plasma membrane where its kinase activity is activated by as yet undetermined mechanisms. Here Ras is modeled as Raf kinase, as implemented by other published computational models. The active phosphorylated Raf subsequently dual phosphorylates and activates MEK1/2 which in turn dual phsphorylates and fully activates ERK1/2 in the activation loop. Phosphatases PP2A and MAP kinase phosphatase 3 (MKP3) are predominantly localized in the cytoplasm and are dual specificity phosphatases capable of dephosphorylating bisphophorylated MEK1/2 and ERK1/2, respectively, in distributive manner.

***(c) KSR-dependent activation of Raf-MEK-ERK signaling on plasma membrane***

***(c1) KSR-mediated Raf-MEK-ERK signaling***

Activated GTP-bound Ras can also recruit downstream Raf, MEK, and ERK kinases to the scaffold protein KSR targeted to the plasma membrane, followed by a series of reactions resulting in ERK activation. In the chemical equations, the KSR-kinase complexes are represented as “KSR” and three numbers representing the state of the signaling molecule on each site: unbound (denoted as 0), inactive state (denoted as 1), active state (denoted as 2). There are two isoforms for MEK and ERK (denoted as a, b). So there are 3*6*6=108 total states, as illustrated in **Figure 2**. Transitions among different scaffold complex species can be caused by binding, unbinding and phosphorylation of kinase molecules. There is no dimerization of scaffold proteins in the model.

***(c2) Regulation of KSR***

There are fine tuning mechanisms that regulate scaffold proteins’ interaction with particular partners. And here, the function of scaffolds KSR and MP1 are mainly regulated by localization, contributing to compartmentalized MAPK signaling. Scaffold protein KSR1 is translocated to the cell membrane in response to growth factor [1]. In quiescent cells, KSR1 exists as KSR1–MEK–PP2A-(p14-3-3)-IMP in cytosol; Upon signal stimulation, KSR1-MEK is released to the plasma membrane.

***(d) Endocytosis, internalization, and recycling of EGFR***

Ligand-dependent receptor internalization is one of the important processes in regulating signal duration and propagation to prevent overstimulation. Upon ligand-induced activation of EGFR, Cbl (a ubiquitin ligase) binds to the activated receptor followed by recruitment of CIN85 (Cbl-interacting protein of 85 kDa) and Endophilin A1. Endophilin A1 is the regulatory component of clathrin-coated pits that binds to lipid bilayers and induces membrane curvature in the early stages of endocytosis. In our model the preformed Cbl-CIN85 complex binds to EGFR dimers followed by Endophilin A1 and is subsequently internalized and degraded. The reversed reactions are assumed as receptor recycling processes.

***(e) Signaling from internalized EGFR to Raf on late endosomes***

Subcellular localization and trafficking play a critical role in coordination of the duration and the specificity of signaling processes. Previous studies in living cells [2,3] demonstrate that Grb2 is also recruited to EGFR on endosomes either directly or through binding to Shc, leading to activation of Raf. Due to a lack of kinetic data, we simplify it as the same series of reactions happening and the same initial concentration of these proteins as in the cytosol near late endosomes as those near the plasma membrane, only the activated EGF receptor here is recycled through endocytosis and internalization.

***(f) MP1-dependent activation of Raf-MEK-ERK signaling on late endosome***

***(f1) MP1-mediated Raf-MEK-ERK signaling***

Endosome-specific scaffold protein MP1/p14 can help the activated, phosphorylated Raf recruit the downstream MEK1 and ERK1 kinases to late endosomes, leading to activation of ERK1. In the chemical equations the MP1-kinase complexes are represented as “MP1” followed by two numbers representing the states of the signaling molecules on each site: unbound (0), inactive state (1), active state (2). Only MEK1 and ERK1 participate, so there are 3*3=9 total states, as illustrated in **Figure 3.** The characteristics of scaffold protein-mediated reactions can be captured through transitions among different scaffold-kinase complex species.

***(f2) Regulation of MP1***

MP1 functions synergistically with spatial regulator p14 and p18 and specifically targets MEK1 and ERK1 to late endosomes [4]. Depending on the concentration of MP1, MAPK signaling is either stimulated or inhibited, which is typical for scaffold protein. However, overexpression of p14 results in concentration-dependent stimulation of MAPK signaling, will not inhibit signaling [5]. Thus, it can be speculated that P14 does not disrupt the formation of signaling modules. In our model here, it is simulated such that P14 and P18’s binding will not affect MP1’s tethering function for MEK1 and ERK1, but only affect the activation reaction within the scaffold.

***(g) Activation and inactivation of PI3K-Akt Component***

Activated EGFR recruits, phosphorylates, and activates PI3K. The active phospho-PI3K phosphorylates PIP2 to PIP3, a second messenger required for activation of Akt (protein kinase B). PDK1 is a kinase that phosphorylates and activates the Akt-PIP3 complex. The active phospho-Akt-PIP3 complex inhibits the activation of Raf1 by hyperphosphorylation on Ser-259 creating a binding site for 14-3-3 protein [6]. The active phospho-PI3K and phospho-Akt-PIP3 are inactivated via dephosphorylation by phosphatases TP4 and Takt, respectively [7].

***(h) The Activation and Inactivation Cycle of RhoA***

The Rho GTPase cycle is subject to tight regulation. Guanine nucleotide dissociation inhibitors (GDIs) maintain Rho proteins in an inactive state in the cytoplasm; guanine nucleotide exchange factors (GEFs) exchanging the bound GDP to the more abundant GTP, and GTPase activating proteins (GAPs) enhance the low intrinsic GTPase activity of Rho family members, thus promoting their inactivation. GDIs, GEFs, and GAPs are themselves tightly controlled by other effectors, and the balance of their activities results in the overall Rho activity.

**References**

1. Kolch W (2005) Coordinating ERK/MAPK signalling through scaffolds and inhibitors. Nat Rev Mol Cell Biol 6: 827-837.

2. Sorkin A, McClure M, Huang F, Carter R (2000) Interaction of EGF receptor and grb2 in living cells visualized by fluorescence resonance energy transfer (FRET) microscopy. Curr Biol 10: 1395-1398.

3. Jiang X, Sorkin A (2002) Coordinated traffic of Grb2 and Ras during epidermal growth factor receptor endocytosis visualized in living cells. Mol Biol Cell 13: 1522-1535.

4. Nada S, Hondo A, Kasai A, Koike M, Saito K, et al. (2009) The novel lipid raft adaptor p18 controls endosome dynamics by anchoring the MEK-ERK pathway to late endosomes. EMBO J 28: 477-489.

5. Teis D, Wunderlich W, Huber LA (2002) Localization of the MP1-MAPK scaffold complex to endosomes is mediated by p14 and required for signal transduction. Dev Cell 3: 803-814.

6. Zimmermann S, Moelling K (1999) Phosphorylation and regulation of Raf by Akt (protein kinase B). Science 286: 1741-1744.

7. Kiyatkin A, Aksamitiene E, Markevich NI, Borisov NM, Hoek JB, et al. (2006) Scaffolding protein Grb2-associated binder 1 sustains epidermal growth factor-induced mitogenic and survival signaling by multiple positive feedback loops. J Biol Chem 281: 19925-19938.
